# Aging is associated with a systemic length-associated transcriptome imbalance

**DOI:** 10.1038/s43587-022-00317-6

**Published:** 2022-12-09

**Authors:** Thomas Stoeger, Rogan A. Grant, Alexandra C. McQuattie-Pimentel, Kishore R. Anekalla, Sophia S. Liu, Heliodoro Tejedor-Navarro, Benjamin D. Singer, Hiam Abdala-Valencia, Michael Schwake, Marie-Pier Tetreault, Harris Perlman, William E. Balch, Navdeep S. Chandel, Karen M. Ridge, Jacob I. Sznajder, Richard I. Morimoto, Alexander V. Misharin, G. R. Scott Budinger, Luis A. Nunes Amaral

**Affiliations:** 1 Department of Chemical and Biological Engineering, Northwestern University, Evanston, IL USA; 2 Northwestern Institute on Complex Systems, Northwestern University, Evanston, IL USA; 3 Center for Genetic Medicine, Northwestern University, Evanston, IL USA; 4 Department of Molecular Biosciences, Northwestern University, Evanston, IL USA; 5 Division of Pulmonary and Critical Care Medicine, Northwestern University, Evanston, IL USA; 6 Simpson Querrey Lung Institute for Translational Science at Northwestern University (SQLIFTSNU), Evanston, IL USA; 7 Department of Biochemistry and Molecular Genetics, Northwestern University, Evanston, IL USA; 8 Department of Neurology, Northwestern University, Evanston, IL USA; 9 Faculty of Chemistry, University of Bielefeld, Bielefeld, Germany; 10 Division of Gastroenterology and Hepatology, Northwestern University, Evanston, IL USA; 11 Division of Rheumatology, Northwestern University, Evanston, IL USA; 12 The Scripps Research Institute, La Jolla, CA USA; 13 Rice Institute for Biomedical Research, Northwestern University, Evanston, IL USA; 14 Department of Physics and Astronomy, Northwestern University, Evanston, IL USA

**Keywords:** Ageing, Systems biology, Data integration

## Abstract

Aging is among the most important risk factors for morbidity and mortality. To contribute toward a molecular understanding of aging, we analyzed age-resolved transcriptomic data from multiple studies. Here, we show that transcript length alone explains most transcriptional changes observed with aging in mice and humans. We present three lines of evidence supporting the biological importance of the uncovered transcriptome imbalance. First, in vertebrates the length association primarily displays a lower relative abundance of long transcripts in aging. Second, eight antiaging interventions of the Interventions Testing Program of the National Institute on Aging can counter this length association. Third, we find that in humans and mice the genes with the longest transcripts enrich for genes reported to extend lifespan, whereas those with the shortest transcripts enrich for genes reported to shorten lifespan. Our study opens fundamental questions on aging and the organization of transcriptomes.

## Abstract (web summary)

The authors found that, across tissues and in multiple datasets, aging is accompanied by a length-associated transcriptome imbalance. In most cases, a decrease in the relative abundance of long transcripts was observed and could be reversed by interventions targeting aging.

## Main

The transcriptome responds rapidly, selectively, reproducibly and strongly to a wide variety of molecular and physiological insults experienced by an organism^1^. While the transcripts of thousands of genes have been reported to change with age^2^, the magnitude by which most transcript levels change is small in comparison with classical examples of gene regulation^2,3^. We hence hypothesize that aging is associated with a phenomenon that affects the transcriptome in a subtle but global manner that goes unnoticed when focusing on the changes in expression of individual genes. Specifically, the small magnitudes of change for individual genes open the possibility that analyses requiring the transcript levels of individual genes to reach specific statistical significance thresholds might not be able to discern statistically significant global changes.

Supporting the perspective that changes may occur at a global level, several studies on animals have reported that RNA formation decreases during aging^4–9^. Moreover, Vermeij et al.^10^ suggested for mice and humans that age-dependent DNA damage leads to a reduction in the expression of long genes by inferring with transcriptional elongation. In contrast to this global perspective, many studies report that transcription factors and microRNAs (miRNAs) can also mediate age-dependent change of transcripts^11,12^. While both global and gene-centric processes contribute to age-dependent changes, it remains unclear which one dominates.

## Results

### Gradient-boosting regression of transcriptomic changes

To avoid potential ambiguity about experimental or analytical choices within datasets from published studies, we performed RNA sequencing (RNA-seq) to measure and survey the transcriptome of 17 tissues from C57BL/6J mice raised under standardized conditions and provided from the colonies of the National Institute on Aging (NIA). We collected data on male mice of 4, 9, 12, 18 and 24 months of age. For every age, we considered six mice, except for rare occasions where a mouse died before data collection, or sample preparation failed for experimental reasons (see Supplementary Table 1 for the number of mice for each tissue and age).

We defined age-dependent transcriptional changes of an individual gene as the fold change of its transcript abundance, which in turn we measured as the log_2_-transformed ratio of the signal attributed to transcripts of one gene at a given age relative to the signal attributed to transcripts of that gene in the same tissue of 4-month-old mice. As total RNA abundance changes for several tissues and cell types during the lifespan of animals^4–9^, it is important to point out that most transcriptomic studies—including ours—included an implicit normalization of the abundance of one transcript relative to all other transcripts. Hence, an observed fold increase for a transcript could still correspond to a lower number of transcript molecules if the molarity of transcripts was reduced for most genes. To explicitly acknowledge this normalization, we used the terms ‘relative fold increase/decrease’ and ‘relative fold change’ instead of the more commonly used terms ‘fold increase/decrease’ and ‘fold change’. To avoid introducing assumptions about the dynamics of temporal changes of transcripts with aging, throughout the paper we considered pairwise comparisons between a given age and 4-month-old mice.

We used a machine learning approach to identify molecular features associated with the relative fold change with age of every protein-coding gene of mice (Supplementary Fig. 1). To be comprehensive, we considered 2,236 broadly cataloged^13,14^ features of individual genes and transcripts. Of these, 310 corresponded to transcription factor binding sites that have been validated in at least one genome-wide assay^14^, whereas 1,912 corresponded to predicted miRNA binding sites^15^. Lastly, 14 features corresponded to architectural properties of genes or transcripts such as the number of exons, guanine–cytosine (GC) content, chromosome number, the number of alternate transcripts and the length of the gene and mature transcripts (Supplementary Table 2).

We used gradient-boosting regression^16^ because it is widely regarded to avoid over-fitting and does not require the amounts of data needed for deep learning approaches. Briefly, gradient-boosting regression creates ensembles of decision trees where optimal criteria for the branching of the tree are determined by the features considered. Gradient boosting iteratively adds decision trees to the ensemble so that the difference between observed changes and changes inferred by the ensemble decreases. We quantified the difference between observed and inferred changes using a Huber loss function^17^, which for a given total absolute distance will favor those ways of branching the trees where the distance will arise from many genes having a small distance while disfavoring those ways of branching the trees where the distance would arise from a few genes having a large distance. This is called the model training step.

**Extended Data Fig. 1:**
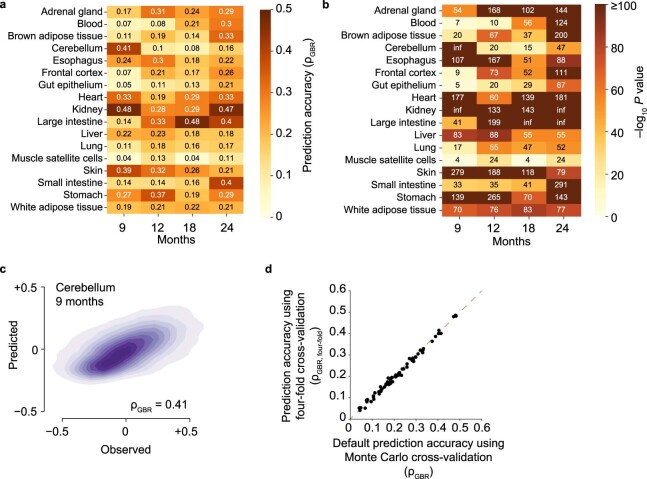
Performance of Gradient Boosting Regression (GBR) models, related to Fig. 1. (**a**) We defined prediction accuracy (ρ_GBR_) as the Spearman correlation between observed and predicted relative fold-changes. (**b**) Significance of the prediction accuracy using t-distribution for two-sided significance test for Spearman correlation^66^. (**c**) Density map of scatter plot of observed and predicted relative fold-changes obtained from the cerebella of 9-months old mice. There is clear correlation between the predicted and observed relative fold-changes, which we quantify through the GBR prediction accuracy (ρ_GBR_). (**d**) Comparison of prediction accuracy following Monte Carlo cross-validation, the default cross-validation scheme of this manuscript, against predication accuracy following four-fold cross-validation for individual combinations of tissues and ages (black dots). In Monte Carlo cross-validation a given gene will on average be used nine times for developing the gradient boosting regression, and on average once for quantifying its performance. In four-fold cross-validation each gene will be considered exactly three times for developing the gradient bossing regression, and exactly once for quantifying its performance.

For every unique tissue–age pair, we created ten gradient-boosting regression models with each model randomly sampling 90% of all genes. We then evaluated the prediction accuracy for every tissue–age pair by applying the trained model to the 10% of genes excluded from the training steps. We evaluated the prediction accuracy as the Spearman correlation between observed and median predicted relative fold changes. Note that the evaluation of the prediction accuracy of the gradient-boosting regression (Extended Data Fig. 1a–c) remained unchanged when following an alternative procedure to sample genes (Extended Data Fig. 1d). Further, the prediction accuracy of gradient-boosting regression matched the experimental accuracy between two cohorts of mice, which each consist of three mice euthanized on different days (Methods and Supplementary Fig. 2).

**Extended Data Fig. 2:**
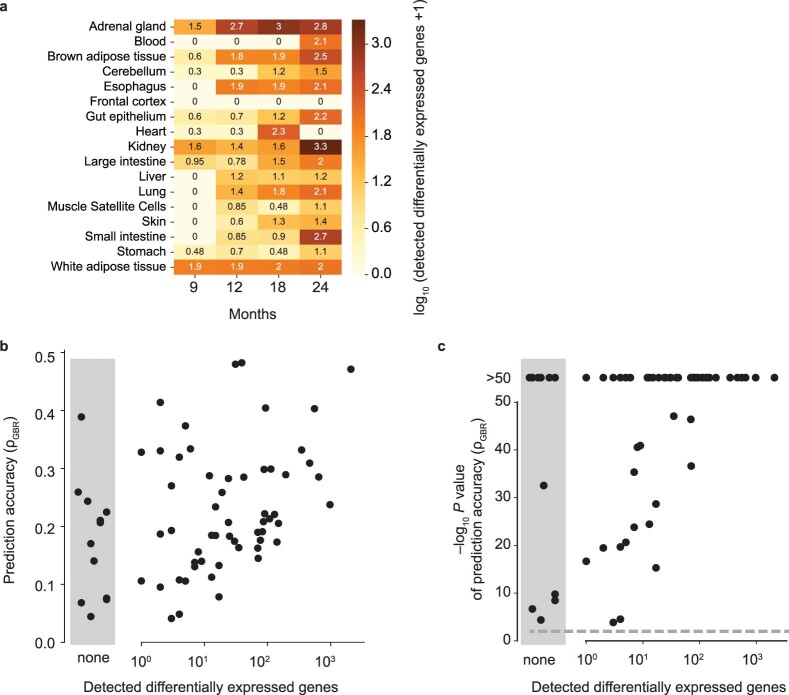
Gradient boosting regression prediction provides good accuracy even when no genes are detected as significantly differentially expressed, related to Fig. 1. (**a**) Number of differentially expressed genes relative to 4-month-old tissues detected at a false discovery rate of 0.05. Values shown are log_10_ (detected differentially expressed genes + 1). For example, a value of 1 in the figure indicates that 9 genes were detected to be differentially expressed. (**b**) Relationship between performance of the prediction accuracy (ρ_GBR_) and the number of individual genes detected as differentially expressed at a false discovery rate of 0.05 across all tissue-age pairs. Reassuringly, we find a positive correlation between the two measures indicating that the ability to predict relative fold-changes is related to there being more genes differentially expressed. Shaded grey area indicates tissue-age pairs without any gene that is being detected as differentially expressed. (**c**) As panel **b**, but showing the significance of correlation between number of differentially expressed genes and the prediction accuracies instead of the prediction accuracy (ρ_GBR_). Significance is determined using t-distribution for two-sided significance test for Spearman correlation^66^.

Lastly, we compared gradient-boosting regression against differential gene expression analyses^2^ and found both approaches to be in good agreement, but gradient-boosting regression was more sensitive (Methods and Extended Data Fig. 2).

### Transcript lengths explain transcriptomic changes

Gradient-boosting regression not only yields predictions about the relative fold change of transcripts but also informs on the importance of individual features to the accuracy of those predictions. We are thus able to return to our initial motivation to identify molecular features associated with the relative fold change of every protein-coding gene.

**Fig. 1:**
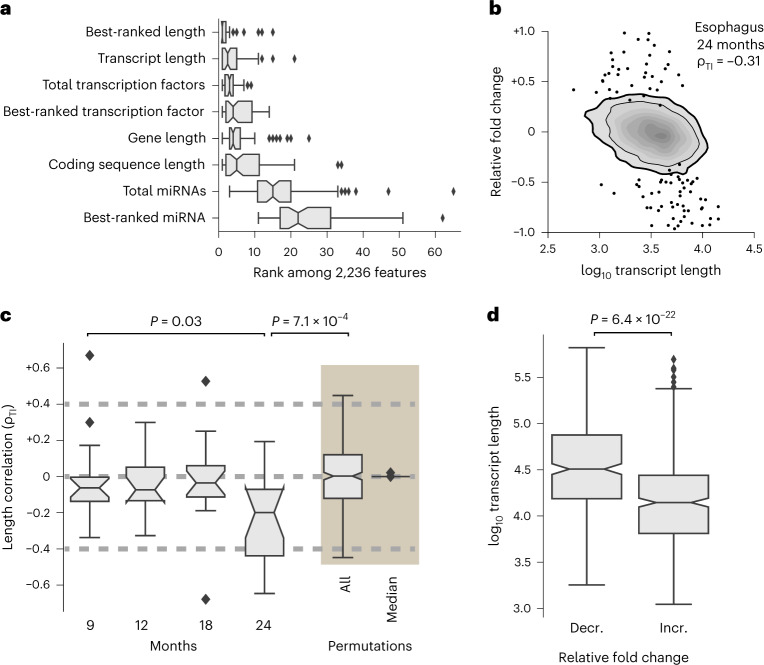
Observation of length-associated transcriptome imbalances. **a**, Importance of individual features or of the best-ranked feature within one group of related features in the gradient-boosting regression across *n* = 68 tissue–age pairs examined over one independent experimental series. **b**, Association between log_10_ transcript length and the relative fold change in transcript abundance between esophagus samples from 4-month-old and 24-month-old mice. We quantified the observed length correlation using the Spearman correlation between transcript length and relative fold changes (ρ_TI_). Kernel density estimates use a linear gray scale. The thin and thicker black lines indicate outermost boundaries of 80% and 90% of kernel density estimates, respectively. Black circles highlight those genes with a relative fold decrease (58) and relative fold increase (46) identified by gene-specific differential expression at a Benjamini–Hochberg-adjusted *P* value below 0.05 with the latter *P* value obtained by DESeq2, which uses a two-sided Wald test. **c**, Box plot of the 68 ρ_TI_ obtained from age comparisons across the *n* = 17 tissues and four ages examined over one independent experimental series. To provide a baseline for individual sample variability, we used permutation testing (beige shading) by assigning mice of the same age to two groups and then obtaining ρ_TI_ between them. ‘All’ marks all *n* = 330 such permutations from data across all 17 tissues and median per tissue. *n* = 17 tissues were examined over one independent experimental series. **d**, NanoString analysis with a metabolism panel to analyze relative fold changes in large-intestine samples gathered from six 24-month-old and six 4-month-old mice. ‘Decr.’ and ‘incr.’ refer to genes with a relative fold decrease and relative fold increase of their transcripts, respectively. We found *n* = 343 genes with a relative fold decrease and *n* = 420 genes with a relative fold increase of their transcripts over one independent experiment. In the box plots (**a**, **c** and **d**), the center is the median, notches indicate the bootstrapped 95% confidence intervals of the median, bounds of the box represent the 25% and 75% percentiles, whiskers extend up to 1.5 times the height of the box, and minima and maxima are the observed minima and maxima. In **c** and **d**, we estimated *P* values using two-sided Mann–Whitney *U* tests (Extended Data Figs. 1–5, Supplementary Figs. 1–8 and Supplementary Tables 1 and 2).

Among the 2,236 considered features, we found that the most consistently informative feature is the median length of mature transcript molecules (Supplementary Table 2), followed by the number of transcription factors, the length of the gene and the median length of the coding sequence (Fig. 1a and Supplementary Figs. 3 and 4). Note that we started assembling the features before the first publication reporting on the association between aging and transcriptional elongation^10^.

**Extended Data Fig. 3:**
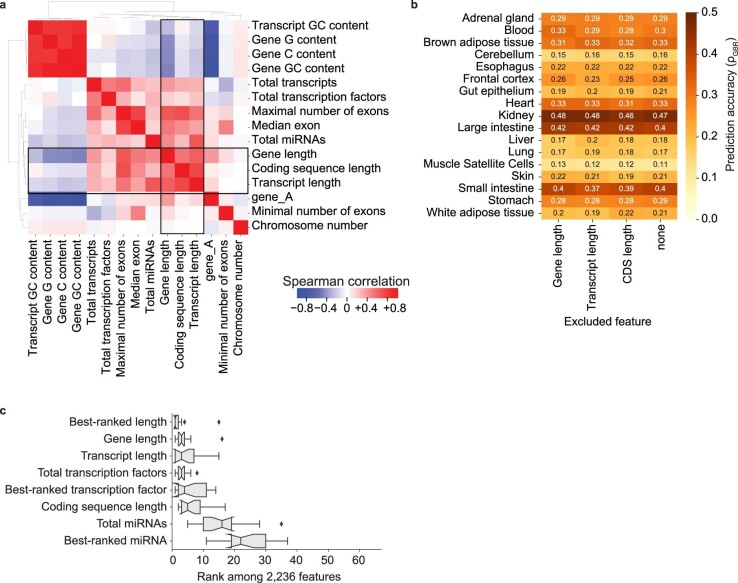
Length-related features contain highly similar information toward age-dependent changes of the mouse transcriptome, related to Fig. 1. (**a**) Pairwise Spearman correlation between selected features. (**b**) Prediction accuracy of relative fold-change for samples from tissues of 24-month-old animals after excluding, in turn, gene length, transcript length, coding sequence (CDS) length, or no feature. The similarity of results confirms that all three length-related features provide the same level of information. (**c**) Importance of individual features or of the best-ranked feature within one group of related features in the gradient boosting regression models across n=17 tissues from 9-months-old mice. In boxplots center is median, notches bootstrapped 95% confidence interval of median, bounds of box 25% and 75% percentiles, whiskers extend height of box 1.5 times, minima and maxima observed minima and maxima.

Features relating to length—namely, the length of the mature transcript, the length of the gene or the length of the coding sequence—were correlated (Extended Data Fig. 3a). These 2,236 features do not include the length of primary transcripts because typically it is not well defined within the GenBank sequence database. Among the 68 tissue–age pairs (17 tissues multiplied by 4 age comparisons to 4-month-old animals), 63 pairs had at least one of the features relating to length among the top 5 explanatory features (Fig. 1a). Demonstrating redundancy across distinct features describing length, omission of any of these features did not affect model performance (Extended Data Fig. 3b).

Note that length-related features are more informative than features describing the binding of any specific transcription factor or miRNA (Fig. 1a). Transcript length also was the most informative feature for the earliest age comparison possible with our experimental survey, 9-month-old versus 4-month-old animals (Extended Data Fig. 3c), hinting at a process with an early onset.

To determine whether transcript length could directly associate with age-dependent changes of the transcriptome, or whether transcript length informs on age-dependent transcriptional change through combinatorial interactions with transcription factors, miRNAs or other architectural features of the genome, we directly compared observed relative fold changes against transcript length.

**Extended Data Fig. 4:**
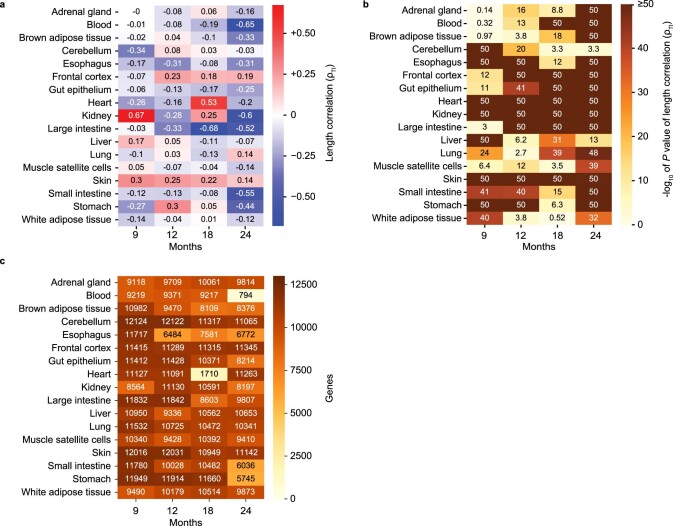
Length-associated transcriptome imbalance for individual tissue-age pairs, related to Fig. 1. (**a**) Length correlations (ρ_TI_) are defined as Spearman correlation between median transcript length and relative fold-changes observed during aging. Note how most length correlations are negative. (**b**) Significance of correlation of panel **a**. Displayed significance values are cropped at 10^−50^. Note how most P values are lower than 10^−20^. Significance is determined using t-distribution for two-sided significance test for Spearman correlation^66^. (**c**) Number of detected genes underlying the computed length correlations (ρ_TI_). Note how in nearly all cases, the correlation was obtained using more than 5,000 protein-coding genes.

We used the Spearman correlation to create a global, genome-wide metric that considers all detected transcripts instead of focusing on a subset of individual genes. We defined the length correlation (ρ_TI_) as the Spearman correlation between transcript lengths and relative fold changes. We found that this global age-dependent transcriptional change extends beyond individual genes identified to be differentially expressed (at adjusted *P* < 0.05) when considered in isolation (Fig. 1b). When comparing transcriptomes of 9-month-old tissues against those of 4-month-old tissues, 10 of 17 tissues already demonstrated a statistically significant anticorrelation (*P* < 0.01; Fig. 1c and Extended Data Fig. 4). The number of affected tissues then remained statistically indistinguishable in 12-month-old and 18-month-old tissues (see 95% confidence intervals of medians as indicated by notches in Fig. 1c). For 24-month-old animals, 14 of 17 different tissues displayed a statistically significant anticorrelation between transcript lengths and relative fold changes (*P* < 0.01; Fig. 1c and Extended Data Fig. 4) and the remaining 3 of 17 different tissues displayed a statistically significant positive correlation between transcript lengths and relative fold changes (*P* < 0.01; Fig. 1c and Extended Data Fig. 4). Thus, the relative abundance of transcripts from long genes can change compared to those from short genes for old mice. To emphasize the systemic nature of the association between transcript length and relative fold changes, we termed this phenomenon ‘length-associated transcriptome imbalance’.

**Extended Data Fig. 5:**
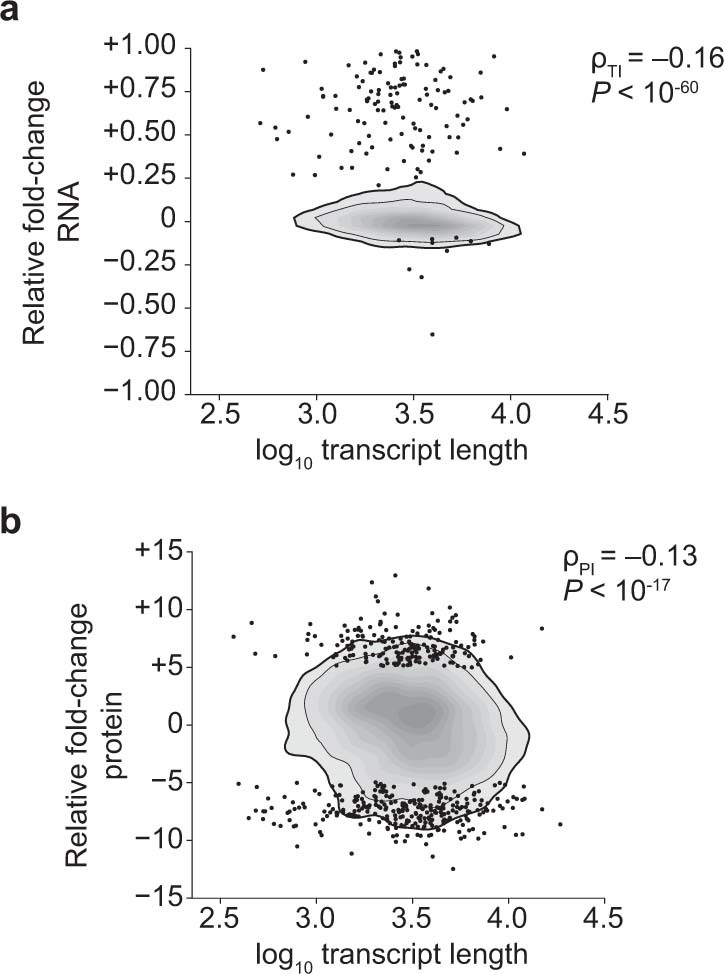
Age-dependent length correlation persists in transcriptomic and proteomic data of Takemon et al.^18^, related to Fig. 1. (**a**) Relative fold-change of age-dependent transcript change as reported by Takemon et al.^18^ who integrate information of transcriptomic change in kidneys of 12- and 18-month-old mice versus 6-month-old mice. Length correlations (ρ_TI_) describes Spearman correlation between median transcript length, and relative fold-change of transcripts. We highlight with black circles those 10 genes with a relative fold-decrease and 186 genes with a relative fold-increase identified by an adjusted *P* value below 0.01 provided by the authors, who obtained *P* values from DESeq2 using a likelihood ratio test. (**b**) Relative fold-change of age-dependent protein change as reported by Takemon et al.^18^ who integrate information of proteomic change in kidneys of 12- and 18-month-old mice versus 6-month-old mice. Proteome length correlations (ρ_PI_) describes Spearman correlation between median transcript length, and relative fold-change of proteins. The thin and thicker black lines indicate outermost boundaries of 80% and 90% of kernel density estimate, respectively. We highlight with black circles those 292 genes with a relative fold-decrease and 186 genes with a relative fold-increase identified by an adjusted *P* value below 0.01 provided by the authors, who obtained *P* values from DESeq2 using a likelihood ratio test.

Before attempting a more detailed biological investigation of the length-associated transcriptome imbalance in mice and other vertebrates, we first performed a detailed interrogation of its robustness. This interrogation showed that the imbalance can alternatively be quantified by the difference in length in the median transcript length of genes with a statistically significant relative fold increase of their transcripts (adjusted *P* < 0.05) and the median transcript length of genes with a statistically significant fold decrease of their transcripts (adjusted *P* < 0.05; Supplementary Fig. 5). We could not find evidence for potential technical artifacts related to the quality of sequencing, the choice of data analysis pipeline, the induction of stress-response genes, inter-animal variability, or whether we consider the length of the transcripts or the genes or the coding sequence (Methods, Fig. 1c and Supplementary Figs. 6–8). Finally, and most importantly, we directly confirmed our main finding of an age-related anticorrelation between transcript length and relative fold changes by means of two experimental approaches other than RNA-seq: NanoString (Fig. 1d) and proteomics (Extended Data Fig. 5)^18^.

### Length association is robust across organisms

To refine the scope of our observation of a length-associated transcriptome imbalance in old mice, we extended our analyses to datasets from transcriptomic studies in mice and other vertebrates from a variety of laboratories^19–22^. To ensure that our findings are representative of earlier reports on aging—and do not reflect upon some idiosyncrasy of our experiments—in our calculations, we used the relative fold changes reported by the authors of the published studies. For studies where relative fold changes for all genes are reported, we quantified the transcriptome imbalance through the length correlation ρ_TI_, defined as the Spearman correlation between transcript lengths and relative fold changes. If only the genes found to have a significant relative fold change of their transcripts were reported, we quantified the transcriptome imbalance by difference in median transcript length of relative fold-increased genes versus relative fold-decreased genes (Supplementary Fig. 5).

**Fig. 2:**
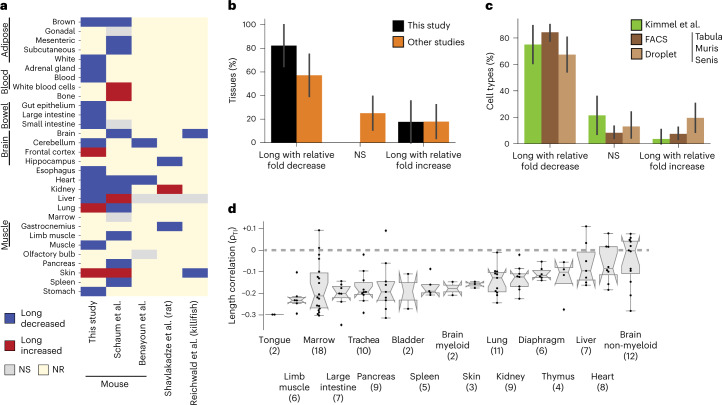
Age-dependent length-associated changes persist across organisms and laboratories. **a**, Presence of a significant length-associated transcriptome imbalance (Methods) in work by Schaum et al.^22^, Benayoun et al.^20^, Shavlakadze et al.^21^ and Reichwald et al.^19^. NR, not registered; NS, not significant. **b**, Fraction of *n* = 17 tissues of this study and *n* = 28 tissues of other studies listed in **a** that showed a significant relative fold decrease of long transcripts or a significant relative fold increase. Tissues were counted for each occurrence in one of the other studies. **c**, Fraction of detected *n* = 28 cell types in Kimmel et al.^23^, and *n* = 121 cell types processed by FACS and *n* = 46 cell types processed using a droplet-based approach (Tabula Muris Senis)^24^ with a fold decrease of long transcripts or a significant relative fold increase. Cell types present in different tissues were counted for each occurrence in one tissue. Comparisons are for each occurrence of a cell type, and relative fold changes observed between an old and a young age group. For Kimmel et al.^23^, the comparison is between 7-month-old and 22- to 23-month-old mice and yielded 28 detected cell types, and for Tabula Muris Senis^24^, between 3-month-old and 24-month-old mice and yielded 121 cell types detected by FACS and 46 cell types detected by droplet-based sequencing. **d**, Box plot of calculated Spearman correlation (ρ_TI_) between log_10_ transcript length and relative fold changes for the different cell types (*n*) reported for individual tissues in Tabula Muris Senis FACS data. In the box plots, the center is the median, notches indicate bootstrapped 95% confidence intervals of the median, bounds of boxes are the 25% and 75% percentiles, whiskers extend up to 1.5 times the height of the box, minima and maxima are the observed minima and maxima. Black dots indicate individual cell types. In **a**–**c**, the presence of an association with length was assessed by a two-sided *t*-distribution significance test of Spearman correlation^66^ at *P* < 0.01. In **b** and **c**, individual data points are not shown as bars represent the fraction of tissues or cell types; thus, error bars are 95% confidence intervals of bootstrapped estimates of these fractions (Extended Data Fig. 6, Supplementary Figs. 9–13 and Supplementary Table 3).

We reanalyzed mouse data from two studies: Benayoun et al.^20^ and Schaum et al.^22^. For the latter, we found that 10 of 17 mouse tissues showed a length-associated imbalance against long transcripts (*P* < 0.01; Fig. 2a and Supplementary Fig. 9a–c). For the former, we found that 2 of 4 mouse tissues showed a length-associated imbalance against long transcripts between 3-month-old and 29-month-old mice (*P* < 0.01; Fig. 2a and Supplementary Fig. 9d). Further, transcripts reported by Benayoun et al.^20^ to change in relative abundance with age independent of any specific tissue showed a length-associated imbalance against long transcripts at the significance cutoff of *P* < 0.05 (Supplementary Fig. 9d).

We also reanalyzed the transcriptome data from rat samples reported by Shavlakadze et al.^21^ for four tissues and compared transcriptome changes between 6-month-old and 24-month-old animals. We found a statistically significant anticorrelation between relative fold changes and transcript length for samples from gastrocnemius and hippocampus tissues (*P* < 0.01; Fig. 2a and Supplementary Fig. 10), and a significant positive correlation for samples of kidney (*P* < 0.01; Fig. 2a and Supplementary Fig. 10a), but no significant correlation for samples of liver (although the latter also showed a significant anticorrelation when considering gene length instead of transcript length; Supplementary Fig. 10b).

Finally, we reanalyzed the transcriptome data from killifish samples reported by Reichwald et al.^19^ and compared transcriptome changes between 5-week-old and 39-week-old animals. We quantified length here as gene lengths, as this was the feature reported by the authors. We found a length-dependent and age-dependent relative fold change in two of three killifish tissues (*P* < 0.01; Fig. 2a and Supplementary Fig. 11).

In their totality, these results support the hypothesis that the tissues of several vertebrate model organisms display an age-dependent transcriptome imbalance and that this imbalance primarily displays a relative fold decrease of long transcripts or genes. The fraction of such tissues that we found in our study (~80%) is comparable (Fisher’s exact *P* = 0.11) to the fraction in other studies (~60%; Fig. 2b).

### Length association is robust across cell types

**Extended Data Fig. 6:**
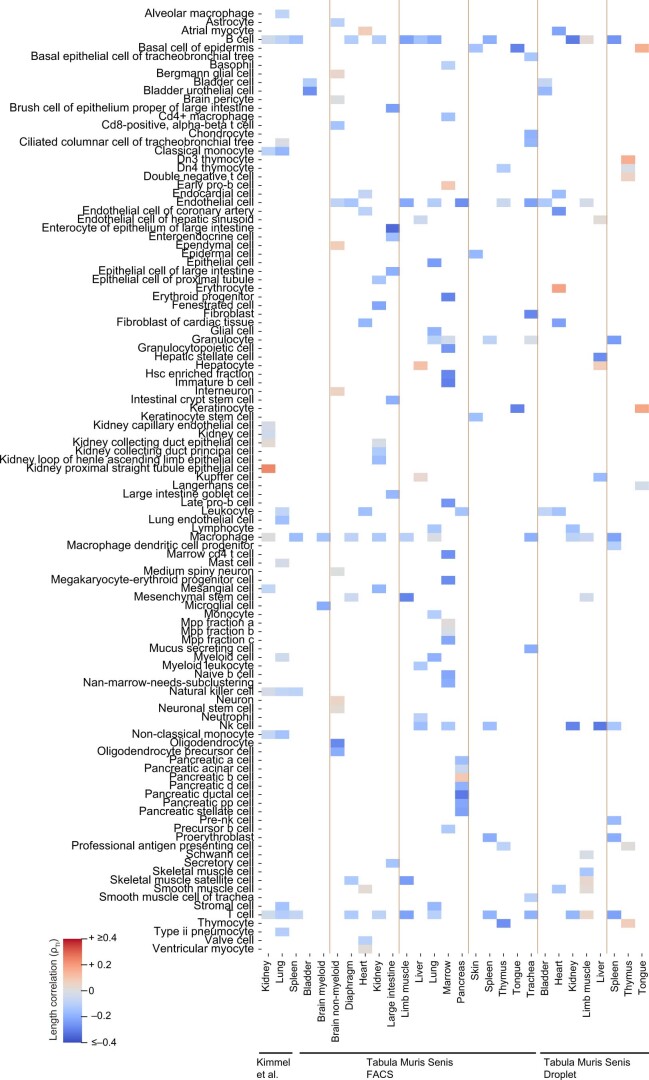
Length correlation with relative fold-changes obtained for individual cell types inferred from single-cell RNA sequencing data, related to Fig. 2. Brown lines are visual guides.

To resolve whether the length-associated imbalance observed among the bulk transcriptomes of entire tissues reflects a change in cellular composition or in the molecular processes occurring in a subset of cell types, we reanalyzed three datasets reporting single-cell transcriptomic measurements during mouse aging. Data reported by Kimmel et al.^23^ enabled us to compare samples from 7-month-old and 22- and 23-month-old mice. Fluorescence-activated cell sorting (FACS)-sorted cells subjected to full-length transcriptome sequencing and, less-sensitive^24^, droplet-based cell isolation paired with sequencing of the 3′ end of transcripts data reported by Tabula Muris Senis enabled to compare samples from 3-month-old and 24-month-old mice. Our reanalysis revealed that most cell types demonstrated a statistically significant anticorrelation between transcript lengths and age-dependent relative fold changes (21 of 28, 102 of 121 and 31 of 46, respectively; Fig. 2c, Extended Data Fig. 6 and Supplementary Table 3). We thus conclude that a length-associated imbalance of the transcriptome occurs in most cell types of mice, and—consistent with our findings for bulk transcriptomes of entire tissues—mainly disfavors long transcripts in aged individuals.

We next explore whether the extent of the length-associated imbalance differed among all detected cell types when comparing individual tissues. We primarily considered FACS-sorted data of Tabula Muris Senis as it provides insight into the largest number of tissues, and then ranked tissues according to the median length-associated transcriptome imbalance of their cell types (Fig. 2d). Of all 136 pairwise comparisons across the different tissues, only 5 comparisons show significant (*P* < 0.01) differences (Supplementary Fig. 12a). This overall absence of statistically detectable tissue-specific differences persisted when considering the other single-cell datasets (Supplementary Fig. 12b–e). Further, it resembled a similar ranking of tissues based on the bulk RNA-seq data generated by us. While it is possible to rank tissues according to the correlation between transcript lengths and age-dependent relative fold changes of transcripts in this bulk RNA-seq data (Extended Data Fig. 4 and Supplementary Fig. 13), biological variability between two cohorts of mice often exceeds observed differences across tissues (Supplementary Fig. 13). We thus conclude that either there are few differences across tissues or current mouse studies do not have sufficient analytical power to reliably detect these differences (Discussion).

### Length-associated transcriptome imbalance in human aging

**Fig. 3:**
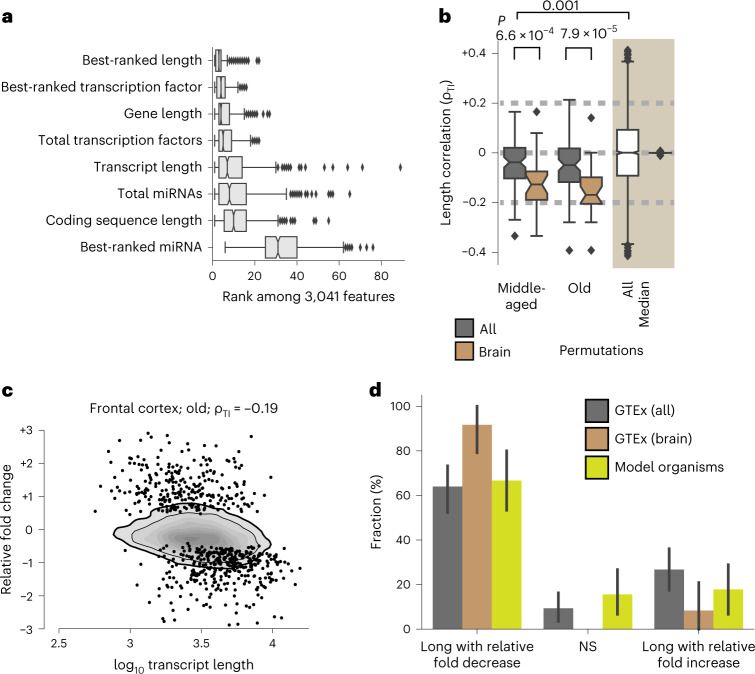
Length-associated transcriptome imbalance in humans. **a**, Importance of features or best-ranked feature of one group of features in the gradient-boosting regression across the *n* = 591 combinations of individual decades, sexes and tissues. **b**, Box plots of the length correalations ρ_TI_ obtained from age comparisons across all *n* = 43 tissues of female donors + *n* = 43 tissues of male donors (dark gray) and across *n* = 12 brain tissues of female donors + *n* = 12 brain tissues of male donors (brown). To provide a baseline for sample variability, shaded in beige, we show the box plot of values of ρ_TI_ obtained from calculating relative fold changes across all permutations of assigning up to six randomly chosen same-age samples to two groups. ‘All’ marks all 2,198 permutations obtained from tissues of male and female donors in the 20- to 29-year and 30- to 39-year age brackets, and ‘median’ indicates the median length-associated changes for *n* = 38 unique tissue in female or male donors. We estimated *P* values with two-sided Mann–Whitney *U* tests. **c**, Relative fold change in transcript abundance in human frontal cortex samples. Kernel-density estimates use a linear gray scale with the thin and thicker black lines being the outermost 80% and 90%, and black circles denote the 349 genes with a relative fold decrease and 266 genes with a relative fold increase by gene-specific differential expression at an adjusted *P* value below 0.05 according to the DESeq2 Wald test. **d**, Extending upon Fig. 2b, the bar plots show the fraction of all (86) and brain-specific (24) tissue–gender pairs of old donors that showed a significant (*P* < 0.01, using *t*-distribution for two-sided significance test for Spearman correlation^66^) relative fold change. *P* values were not corrected for multiple hypotheses. As individual data points cannot be shown as representing the fraction of tissues, error bars are the 95% confidence intervals of bootstrapped estimates of these fractions. Tissues were counted for each occurrence in another studies. In the box plots (**a** and **b**), the center is the median, notches indicate bootstrapped 95% confidence intervals of the median, bounds of boxes are the 25% and 75% percentiles, whiskers extend up to 1.5 times the height of the box, minima and maxima are the observed minima and maxima (Extended Data Fig. 7, Supplementary Figs. 14 and 15 and Supplementary Tables 4–6).

To determine whether a length-associated transcriptome imbalance also occurs in human aging, we next reanalyzed data collected by the Genotype-Tissue Expression (GTEx) consortium^25^ from human tissues at the time of death (Supplementary Fig. 14a). We again identified length-related features were predictive of relative fold change of transcripts (Fig. 3a), with gene length here being the most informative individual feature in the trained models (Fig. 3a and Supplementary Table 4). For consistency with our preceding analyses, however, we focused on transcript length (Fig. 3b,c). Our analysis of human tissues again showed an anticorrelation between transcript length and relative fold changes (Fig. 3b,c and Supplementary Tables 5 and 6). This finding was also recovered when considering either the length of the gene or the length of the coding sequence instead of transcript length (Supplementary Fig. 14b,c).

An anticorrelation between transcript lengths and relative fold changes was already apparent for most tissues from middle-aged donors (40–59 years) in comparison with those from young donors (20–39 years; Fig. 3b). The share of tissues affected (~60%; Fig. 3d) in old donors (60–79 years) was similar to that reported above for the vertebrate model (Fig. 2). We conclude that a length-associated transcriptome imbalance also occurs in humans and that it too preferentially leads to a relative fold decrease of long transcripts.

**Extended Data Fig. 7:**
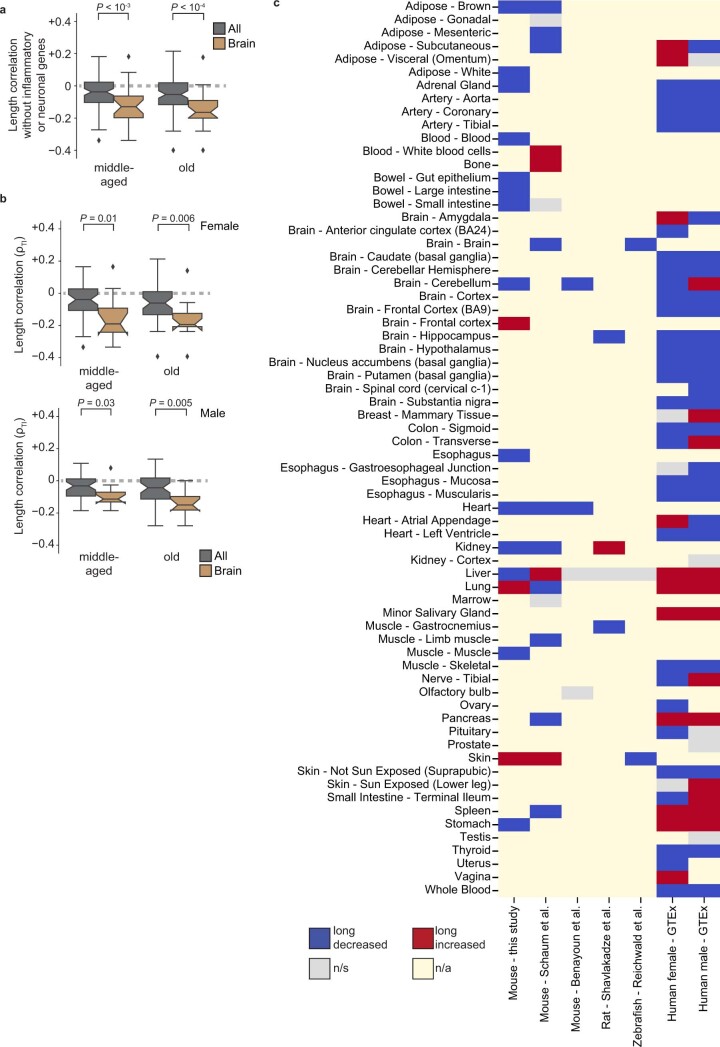
Robustness of observation of length-associated transcriptome imbalance in humans, related to Fig. 3. (**a**) Length correlation after excluding genes annotated for inflammatory and neuronal processes across all 43 tissues of female donors and all 43 tissues of male donors (dark grey) and across 12 brain tissues of female donors and 12 brain tissues of male donors (brown) considered. *P* values represent two-sided Mann Whitney *U* tests with *U* values of 571 and 459 tissues from middle-aged and old donors, respectively. (**b**) We find a length-associated transcriptome imbalance for tissues from female (top) and male donors (bottom). n=median across all 43 tissues of female donors and all 43 tissues of male donors (dark grey) and across 12 brain tissues of female donors and 12 brain tissues of male donors (brown), respectively. In boxplots center is median, notches bootstrapped 95% confidence interval of median, bounds of box 25% and 75% percentiles, whiskers extend height of box 1.5 times, minima and maxima observed minima and maxima. *P* values represent two-sided Mann Whitney *U* tests with *U* values of 136 and 122 for tissues from middle-aged and old female donors and *U* values of 148 and 121 from middle-aged and old male donors, respectively. (**c**) As Fig. 2a but additionally including human tissues.

Given the large number of donors within the GTEx (Supplementary Fig. 14a), we revisited the question of whether the transcriptome imbalance affects all tissues equally. We ranked tissues by the magnitude of their transcriptome imbalance and found that we could reject this null hypothesis (Supplementary Fig. 15) as we observed that the imbalance was significantly (*P* < 0.001) stronger in brain tissues (Mann–Whitney *U* test values of 561 and 486 for tissues from middle-aged and old donors; Fig. 3b and Supplementary Fig. 15); this finding persisted even when excluding genes relating to inflammatory and neuronal processes (Extended Data Fig. 7a) or controlling for gender (Extended Data Fig. 7b,c). This pattern is also consistent with the findings of an independent study on human hippocampus that explicitly demonstrated a relative fold decrease with age of the transcripts from long genes^10^.

### Length association responds to antiaging interventions

**Extended Data Fig. 8:**
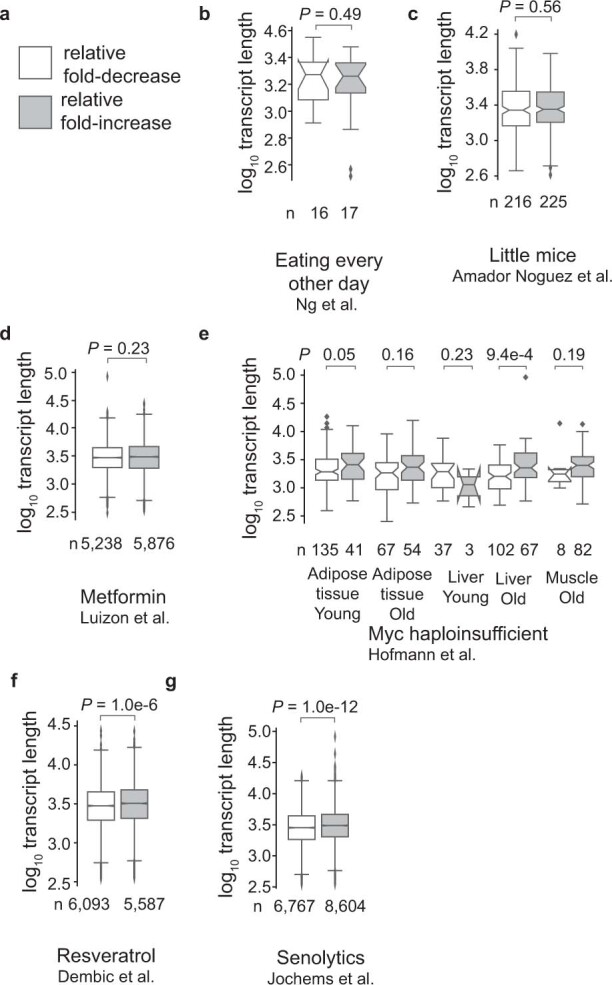
Anti-aging interventions oppose the relative fold-decrease of long transcripts, related to Fig. 4. (a) We use color-coding in all panels to indicate genes transcripts showing a relative fold-decreased (white) or relative fold-increase (grey) according to the authors of the respective studies. (**b**) Transcript length for genes reported by Ng et al.^95^ to be differentially expressed in the liver of mice eating every other day for three months after reaching 3 months of age. (**c**) Transcript length for genes reported to be differentially expressed in Little mice by Amador-Noguez^90^ for the liver in an analysis including 3, 6, 12, and 24-month-old animals. (**d**) Transcript length for genes reported to be differentially expressed by Luizon et al.^96^ following exposure of primary human hepatocytes to metformin. (**e**) Transcript length for genes reported to be differentially expressed in Myc haploinsufficient mice by Hofmann et al.^97^ for indicated tissues and in 5-month-old (young) and 24-month-old (old) mice. Note that for 5-month-old muscle no comparison is given as no gene was reported to show a relative fold-decrease. (**f**) Transcript length for genes reported to be differentially expressed by Dembic et al.^98^ following exposure of human primary fibroblasts to resveratrol. Given the genome-wide readout provided by the authors we could additionally compute *P* values using t-distribution for two-sided significance test for Spearman correlation^66^, which will yield 0.0002. (**g**) Transcript length for genes reported to be differentially expressed by Jochems et al.^99^ following exposure of cultured cells toward at least one senolytic compound. *P* values were estimated by two-sided Mann-Whitney *U* test. n indicates number of genes with a relative fold-decrease and relative fold-decrease of their transcripts. Given the genome-wide readout provided by the authors we could additionally compute *P* values using t-distribution for two-sided significance test for Spearman correlation^66^, which will yield 7.0e-9. (**b-g**) In boxplots center is median, notches bootstrapped 95% confidence interval of median, bounds of box 25% and 75% percentiles, whiskers extend height of box 1.5 times, minima and maxima observed minima and maxima.

To systematically investigate whether the length-associated transcriptome imbalance is related to aging or merely to the passage of time, we considered 11 antiaging interventions with strong support for extending lifespan within the Interventions Testing Program of the NIA^26,27^ and where differential expression results of transcripts were published and directly provided by the authors of the corresponding studies. Within these published differential expression results, we found statistically significant (*P* < 0.01) support for a preferential fold increase of long transcripts for 7 interventions (Fig. 5a–f and Extended Data Fig. 8): fibroblast growth factor 21 (FGF21) excess, Myc heterozygosity, rapamycin, resveratrol, S6 kinase 1 (S6K1) deletion, senolytics and Snell mice. We did not find statistically significant support (*P* > 0.01) for the 4 remaining interventions: Ames mice, eating every other day, Little mice and metformin.

We cannot say whether the absence of a significant length-associated relative fold change for 4 interventions reflects upon the true potential of these interventions or upon experimental parameters, sample size or the number of genes reported by the authors of the original studies. Curiously, among all tested combinations of antiaging interventions and tissues and ages, the incidence rate of a significant (*P* < 0.05) effect seemed somewhat smaller for in vivo than for cell culture settings (7 of 16 and 3 of 4, respectively; two-sided Fisher’s exact test, *P* = 0.58). Given the relative fold increase of long transcripts following 7 of the 11 interventions analyzed by us and preceding results by Vermeij et al.^10^ on caloric restriction opposing the length-associated transcriptome imbalance in an ERCC excision repair 1 (ERCC1) progeroid mouse model, we can conclude that the length-associated transcriptome imbalance is responsive to antiaging interventions and that several interventions promoting longevity oppose the direction of the transcriptome imbalance that we are reporting here to be the primary direction of the relative fold change encountered within vertebrates (Figs. 2b,c and 3d).

**Fig. 4:**
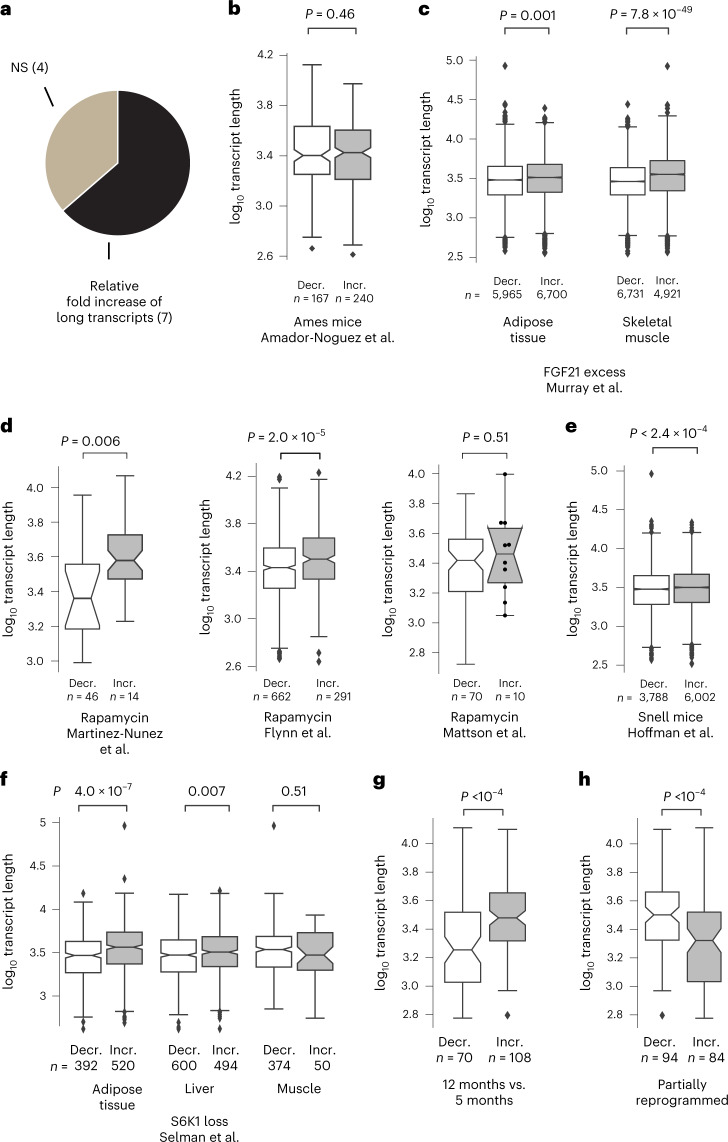
The age-dependent length-associated transcriptome imbalance is responsive to several antiaging interventions. **a**, Share of the 11 antiaging interventions of the Intervention Testing Program^26^ tested that reduced the relative fold decrease of long transcripts with aging. NS, not statistically significant at the *P* < 0.01 level following two-sided Mann–Whitney *U* test. **b**, Transcript length for genes reported to be differentially expressed in Ames mice^90^ for liver in an analysis including 3-, 6-, 12- and 24-month-old animals. **c**, FGF21 excess for adipose tissue and skeletal muscle following exposure of rhesus macaques to FGF21 (ref. ^86^). **d**, Rapamycin findings reported by Martinez-Nunez et al.^91^ for HEK293 cells following exposure, reported by Flynn et al.^87^ for hearts of 24-month-old mice following 3 months of exposure, and reported by Mattson et al.^92^ for cytotoxic T cells following 3 months of exposure. **e**, Transcript length for genes reported to be differentially expressed in Snell mice^93^ for brown adipose tissue. **f**, Deletion of ribosomal S6 protein kinase 1 (S6K1)^94^ for 600-day-old female mice. **g**, Relative fold changes of transcripts in retinal ganglion cells of 12-month-old mice compared to 5-month-old mice^28^. **h**, Retinal ganglion cells of 12-month-old mice following ectopic expression of *Pou5f1*, *Sox2* and *Klf4* (ref. ^28^). In **b**–**h**, the box plots of the lengths of transcripts show a relative fold decrease (decr.) or relative fold increase (incr.) upon exposure (for details of ref. ^87^, see the Methods). In the box plots, the center is the median, notches indicate bootstrapped 95% confidence intervals of the median, bounds of boxes are the 25% and 75% percentiles, whiskers extend up to 1.5 times the height of the box, minima and maxima are the observed minima and maxima. Black dots indicate observations if the number of genes ≤ 10. We estimated *P* values with two-sided Mann–Whitney *U* tests. *n* indicates the number of genes with a relative fold decrease and relative fold increase. The notches display bootstrapped 95% confidence intervals of transcript length (Extended Data Fig. 8).

In addition to the above interventions, we probed rejuvenation by partial reprogramming through ectopic expression of *Pou5f1*, *Sox2* and *Klf4*. We turned to a recent study by Lu et al.^28^, which reported transcriptomic data on retinal ganglion cells in middle-aged 12-month-old mice relative to 5-month-old mice. Surprisingly, but consistent with our observations in a minority of tissues (Fig. 2b,c and Fig. 3d), we found a length-associated transcriptome imbalance with a relative fold increase (rather than fold decrease) of long transcripts during aging (Fig. 4g). However, following rejuvenation, the relative fold increase of long transcripts in 12-month-old mice relative to 5-month-old mice reverted (Fig. 4g,h). We thus conclude that the length-associated transcriptome imbalance is responsive to antiaging interventions.

### Shortest and longest transcripts impact longevity opposingly

To further probe the relation between the observed length-associated transcriptome imbalance and aging, we next examined a previously established catalog of ‘pro-longevity’ and ‘anti-longevity’ genes for which a loss-of-function mutation decreases or increases the lifespan of model organisms, respectively^29^. We mapped the genes listed in the catalog onto their human and mouse orthologs and performed an enrichment analysis considering all protein-coding genes that occurred at least once in the database used for annotation. The identified 665 genes accounted for a small subset of the genome (3%) and, notably, in human tissues showed a nearly indistinguishable length-associated transcriptome imbalance as other genes (Spearman, 0.93; Supplementary Fig. 16a).

**Fig. 5:**
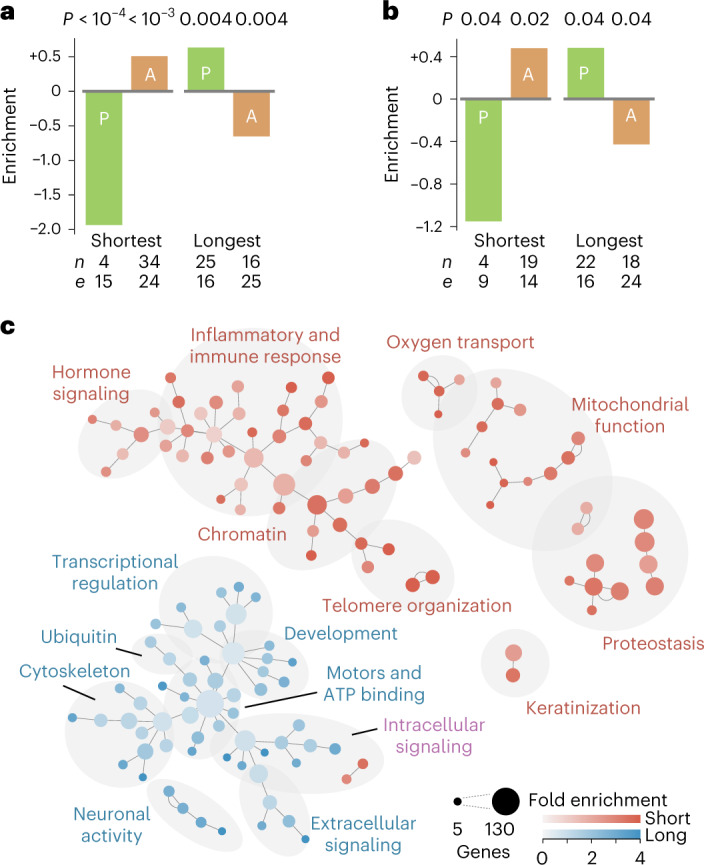
Enrichment analysis suggests that the shortest and longest transcripts have opposing associations with longevity. **a**,**b**, Fold enrichment for ‘pro-longevity’ (P, green) and ‘anti-longevity’ (A, orange) of protein-coding human genes (**a**) or mouse genes (**b**) among the genes with transcript lengths in the bottom 5% or top 5% of lengths. Negative enrichment indicates depletion; *n* indicates the observed number of genes with a pro-longevity or anti-longevity phenotype among these genes with extreme transcript lengths; *e* indicates expected number of genes with a pro-longevity or anti-longevity phenotype if there was no association between transcript lengths and longevity phenotypes. We estimated *P* values using two-sided Fisher’s exact test. The data suggest that pro-longevity genes may be depleted among the shortest genes and may be enriched among the longest genes. **c**, Human Gene Ontology analysis for annotation enrichment among genes with transcripts in the bottom 5% of transcript lengths and annotation depletion among genes with transcripts in the top 5% of transcript lengths. Area of circle is proportional to number of genes. Edges represent highest embedding of a lower-level hierarchical annotation (smaller circle) within a higher-level one (larger circle). Red (blue) indicates genes enriched in genes with shortest (longest) transcripts (*P* < 0.05; Benjamini–Hochberg-corrected Fisher’s exact test; Extended Data Figs. 9 and 10, Supplementary Fig. 16, Supplementary Data 1 and Supplementary Tables 7–10).

**Extended Data Fig. 9:**
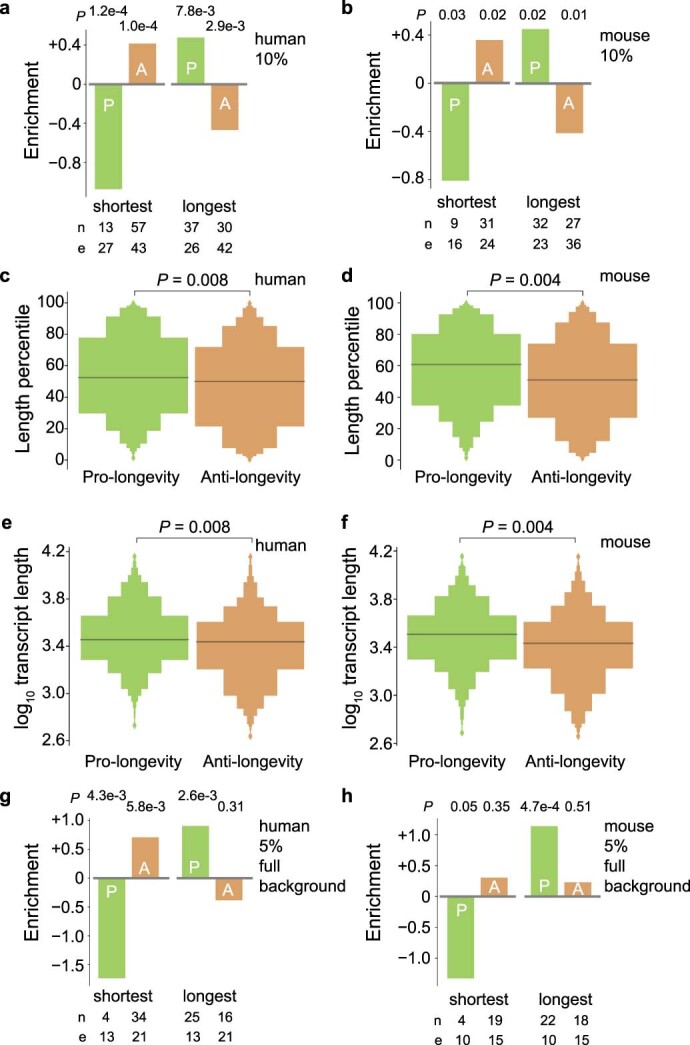
Transcript length and reported association to the biology of aging, related to Fig. 5. (**a**) Fold enrichment for ‘pro-longevity’ (P, green) and ‘anti-longevity’ (A, orange) genes among the genes with the 10% shortest and 10% longest median transcript lengths in humans. Negative enrichment indicates depletion. (**b**) As panel a, but for mice. (**c**) Letter value plot of all 294 pro-longevity and 455 anti-longevity genes across the human genome, with all protein-coding genes ranked according to length and rank transformed to percentile. (**d**) Letter value plot but for all 219 pro-longevity and 335 anti-longevity genes across the mouse genome, with all protein-coding genes ranked according to length and rank transformed to percentile. (**e**) Letter value plot of transcript length of all 294 pro-longevity and 455 anti-longevity genes across the human genome. (**f**) Letter value plot of transcript length all 219 pro-longevity and 335 anti-longevity genes across the mouse genome. (**g**) As Fig. 5a, but using all human protein-coding genes as the set being analyzed instead of restricting enrichment analysis toward genes with at least one annotation and focusing on the 5% shortest and 5% longest median transcript lengths. (**h**) As Fig. 5b, but using all protein-coding genes as the set being analyzed instead of restricting enrichment analysis toward genes with at least one annotation and focusing on the 5% shortest and 5% longest median transcript lengths in mice. (**a,b, g, h**) n indicates the absolute number of genes with a pro-longevity and anti-longevity phenotype among the genes with 5% or 10% (as indicated as focus in pales) shortest and longest median transcript lengths and e indicates expected number of genes with a pro-longevity and anti-longevity phenotype if there was no association between transcript lengths and longevity phenotypes. *P* values were estimated by two-sided Fisher’s exact test. (**c-f**) Letter value plots shows median as black lines, boxes of decreasing width percentiles of data according to letter ratios (+/− 25%, +/−37.5, +/−43.75, etc.), and observed minima and maxima as diamonds. *P* values are from two-sided Mann-Whitney *U* test.

We focused on the genes with the 5% shortest median transcript lengths and the genes with the 5% longest median transcript lengths as those genes are most poised to show a length-associated relative fold change during aging (Supplementary Fig. 16b,c). The enrichment analysis showed that genes encoding the shortest transcripts were significantly depleted from pro-longevity genes and significantly enriched for anti-longevity genes, whereas the genes encoding the longest transcripts were significantly enriched for pro-longevity genes and significantly depleted from anti-longevity genes (Fig. 5a,b). This observation was robust against the specific threshold chosen for transcripts to be classified as short or long (Extended Data Fig. 9a,b) and held when considering differences in median transcript length between pro-longevity and anti-longevity genes (Mann–Whitney *U* test *P* = 0.008 and *P* = 0.004 for human and mouse, respectively; Extended Data Fig. 9c–f). Further, the enrichments and depletions reached significance (*P* < 0.01) for one of four human cases and three of four murine cases when considering all human protein-coding genes instead of only those annotated for longevity phenotypes (Methods and Extended Data Fig. 9g,h).

As cellular pathways can be encoded by genes of different gene lengths^30^, we next investigated whether the shortest and longest transcripts in humans encode cellular processes and physiology that has been associated with aging. To identify candidate processes, we performed a Gene Ontology analysis for annotations enriched among transcripts of one length extreme and simultaneously depleted among transcripts of the other length extreme. We determined the false-positive rate of this approach to identify such annotations. Briefly, we determined the false-positive rate was negligible as no more than one single annotation appeared to be enriched within 200 different randomizations (Methods).

**Extended Data Fig. 10:**
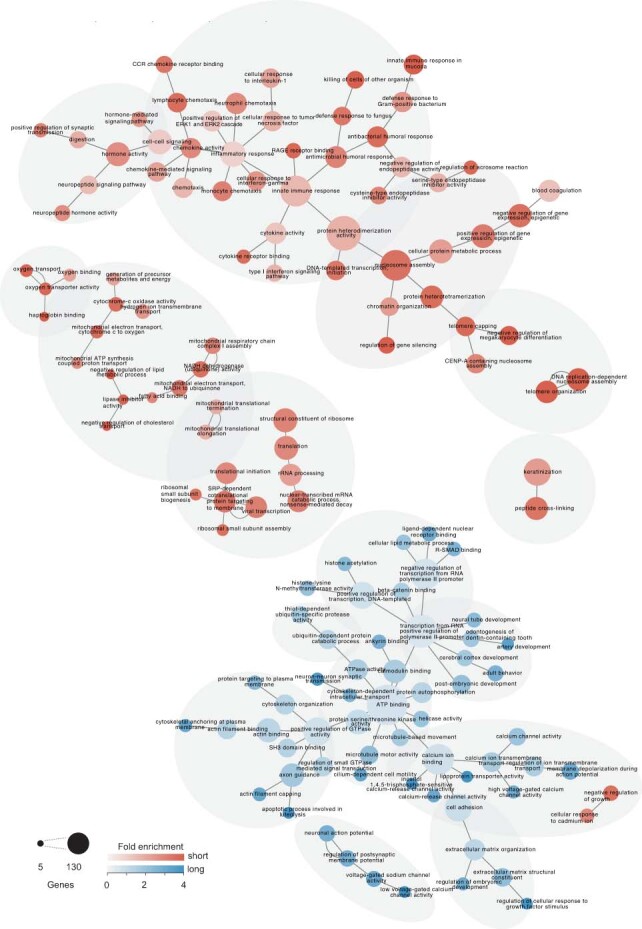
Network of Gene Ontologies enriched for transcripts of one length-extreme and depleted from the other, related to Fig. 5. Human Gene Ontology analysis for annotation enrichment among genes with transcripts in the bottom 5% of gene lengths and annotation depletion among genes with transcripts in the top 5% of gene lengths. Area of circle is proportional to number of genes in set. Edges represent highest embedding of a lower-level hierarchical annotation within a higher level one. Red (blue) indicates genes enriched in genes with shortest (longest) transcripts (*P* < 0.05; Benjamini-Hochberg corrected two-sided Fisher’s exact test).

Upon analysis of the annotations observed within the human genome, we found 138 annotations of mouse genes and 140 annotations of human genes to be enriched among transcripts of one length extreme and simultaneously depleted among transcripts of the other length extreme. Reassuringly, our approach independently recovered well-established observations concerning the molecular, cellular and physiological processes associated with aging (Supplementary Tables 7–10) and partially recapitulated a recent study that only considered the enrichment of individual annotations^31^. We found that the anabolic branches of proteostasis, mitochondrial function, telomere maintenance, chromatin organization and immune function^32,33^ were enriched among the shortest transcripts and depleted from the longest transcripts. Transcriptional regulation^34^, developmental processes^35^, ATP binding^36^, cytoskeletal structure and synaptic activity^37^ were enriched among the longest transcripts and depleted from the shortest transcripts. To help navigation across related annotations, we constructed a network representation that presents individual annotations according to the shared overlap of annotated genes rather than the hierarchical structure provided by the Gene Ontology analysis (Methods, ref. ^38^ and Fig. 5c). See Extended Data Fig. 10 and Supplementary Data 1 for labels of individual Gene Ontology terms within this network. Collectively, these findings demonstrate a remarkably high overlap between the functions encoded by the shortest and longest transcripts and the biological hallmarks of aging^33,39^.

The results presented herein thus strongly support the idea that genes encoding opposing roles toward longevity could be distinctively and systemically affected by the length-associated transcriptome imbalance. In most tissues, the length-associated transcriptome imbalance should thus promote the expression of genes that appear to contribute to aging (Figs. 2b,c and 3d), whereas in some tissues (Figs. 2b,c and 3d), the length-associated transcriptome imbalance should promote the expression of genes that appear to contribute to staving off aging.

## Discussion

We recognize that our study holds a far-reaching implication on how RNA-seq studies are analyzed and interpreted. Technical biases in RNA-seq studies, which affect gene-specific readouts according to their length, have been reported widely, and several tools have been subsequently deployed to computationally counter the effects of this length association^40–43^. As demonstrated by our NanoString experiment, and by our reanalysis of published proteomics data, this data processing step may hide biologically driven associations between transcript length and relative fold change.

Our observational study invites further work on the molecular and temporal onset of the length-associated transcriptome imbalance, the role of gene regulatory networks, population variability in larger cohorts, the normalization of gene expression data during aging and whether an initial length-associated transcriptome imbalance could be causative to aging. Moreover, whether our current findings on aging would extend to further genetic backgrounds and genes whose role in shortening or lengthening lifespan have not yet been discovered^44^.

However, perhaps the most pressing remaining question relates to the origin of the length-associated transcriptome imbalance during aging. Our findings about the genes with the shortest and longest transcripts enriching for genes with different roles toward longevity could be viewed as support for longevity-related roles of genes driving the evolution of their transcript length. However, this explanation would presently only appear to account for a fraction of the genes that show a transcript length-associated change during aging (Figs. 2b,c and 3d). Further, gene lengths appear largely invariant in a phylogenetic kingdom^45,46^ and differences in gene and protein lengths have already been attributed to multiple factors, including cellular energy constraints, expression levels and gene duplications^47,48^.

Turning to earlier literature, a length-associated transcriptome imbalance does not appear specific to aging itself. Moreover, there seem to be multiple potential molecular origins for a length-associated transcriptome imbalance. Most prominent among the specific molecular mechanisms, DNA damage has been explicitly demonstrated to yield a length-associated transcriptome imbalance with a relative fold decrease of the longest transcripts in a progeroid model of aging^10^. Heat shock, which challenges proteostasis, a hallmark of aging^33^, leads to a length-associated transcriptome imbalance by causing premature transcriptional termination through cryptic intronic polyadenylation^49^. Similarly, loss of splicing factor proline/glutamine (*Sfpq*), encoded by the gene that displays the strongest differential splicing during human aging^50^, has been shown to yield a length-associated transcriptome imbalance by interfering with transcriptional elongation of long genes^51^. Methyl CpG binding protein 2 (MeCP2) opposes a length-associated transcriptome imbalance by dysregulating transcriptional initiation according to the length of the gene body^52,53^. Further, patients with Alzheimer’s disease show a length-associated transcriptome imbalance whose onset has been suspected to stem from somatic mutations that affected transcript stability^54^.

Jointly, these observations invite the unsupported hypotheses that during aging there may not be a single origin for the length-associated transcriptome imbalance and that the length-associated transcriptome imbalance in aging instead represents an intermediate step within a ‘bowtie structure’ through which multiple environmental and internal conditions simultaneously affect multiple downstream outputs^55–57^. The length-associated transcriptome imbalance thus may offer itself as an explanation for the recent observation of inter-tissue convergence of gene expression during aging^58^. Further arguing in favor of an integrative role of the length-associated transcriptome imbalance, we find evidence that several distinct antiaging interventions counter the length-associated transcriptome imbalance against long transcripts despite these different antiaging interventions partially affecting different aspects of cellular and organismal physiology^59,60^.

One superficially conflicting observation, which may further help to narrow down the origin of the length-associated transcriptome imbalance, is the realization that in a small subset of tissues and cell types the longest transcripts display a relative fold increase rather than a relative fold decrease (Figs. 2b,c, 3d and 4g), with the former also appearing to be malleable to antiaging interventions (Fig. 4h). One possible explanation is that there exist two independent types of length-associated transcriptome imbalances of which the first one displays a relative fold increase of the longest transcripts, and the second one a relative fold decrease of the longest transcripts. Alternatively, both types of length-associated transcriptome imbalances could be vis-à-vis manifestations of a single phenomenon such as changes in the net processivity of the formation of mature transcripts. Specifically, we suspect that the age-dependent length-associated transcriptome imbalance in vertebrates primarily displaying a relative fold decrease (rather than relative fold increase) of long transcripts may arise from the hypothesis that there are more ways for perturbations to break than to improve cellular function^61^.

As the shortest and longest transcripts enrich for opposing roles toward longevity, opposing types of the length-associated transcriptome imbalance in different tissues also open the questions on whether these tissues are poised differentially toward aging and there could be trade-offs between tissues during aging beyond the recognized trade-offs between somatic and germline tissue^62^. Independent of the specific direction of relative fold changes, altered transcript levels of thousands of genes or of specific subsets of genes may also promote aging by challenging epigenetic, transcriptional and proteomic homeostasis^63,64^ and reducing the capacity of cells to properly respond to internal or external factors (such as protein aggregates or pathogens). Further independent of any transcript length, imbalances in gene expression have the potential to alter subcellular stoichiometries^65^.

Spurred by our findings on antiaging interventions, we believe that understanding the direction of causality between other age-dependent cellular and transcriptomic changes and length-associated transcriptome imbalance could open novel research directions for antiaging interventions.

## Methods

### Statistics and reproducibility

No statistical methods were used to predetermine sample sizes, but our sample sizes are similar to those reported in previous publications^21,22,24^. We performed a two-cohort design to estimate reproducibility after our experiments and focused our analytical approach on the identification of patterns that are detectable within our given sample number. For testing the generalizability of our findings, we considered external datasets in mice and other organisms.

In an initial pilot analysis, we used a Lilliefors test to assess assumptions on normality. For comparing different ages, we used a two-sided Mann–Whitney *U* test to account for non-normality and visually double-checked that compared groups would have a similar skewness. For quantifying the significance of genome-wide correlations (and thus bypassing the need for gene-specific *P* values), we used a *t*-distribution test as the latter appears to be an accurate approximation when working with thousands of data points^66^.

Mann–Whitney *U* test, Spearman correlations and Fisher’s exact test were computed through scipy.stats (version 1.2.1)^67^. Bootstrapped estimates of the 95% confidence intervals of the medians were obtained through Seaborn^68^. For comparison purposes, we also performed differential gene expression analyses using DESeq2 (ref. ^69^), which provide significance values that follow a set of frequent assumptions on gene expression distributions^70^. Significance of the difference between two correlations was tested with Daniel Soper’s Free Statistics Calculators 4.0 (https://www.danielsoper.com/statcalc/), which implemented a corresponding test developed by Fisher^71^.

For adjusted *P* values, we followed a Benjamini–Hochberg correction^72^.

No randomization of samples was performed by us as we had ordered mice of different ages and allocated mice of different ages to different groups for analysis.

Investigators were not blinded to group allocation during data collection and outcome assessment and further data analysis. Blinding during data collection was not possible as old mice look different from younger mice; blinding was not relevant during data analysis, as the latter used a machine learning strategy to find the properties informing on age-dependent change.

No data were excluded from the analyses, except for additional control analysis, which tested the robustness of the conclusion against different exclusion criteria (Supplementary Fig. 10c–f).

After completion of the manuscript, however, we noted that the original experiment contained muscle tissues for which no sequence data were obtained, and that the original preparation of sequence data included sorted alveolar macrophages, alveolar type 2 cells and monocyte-derived dendritic cells. Preceding the analysis started in this paper, sequence data of the latter had not been carried forward toward analysis as quality-control (QC) metrics appeared different and indicative of lower quality than the other experimental preparations. Retrospectively, analyzing these three cell populations toward a length-associated transcriptome imbalance after the (otherwise) completion of this paper, we find, consistent with our comprehensive reanalysis of cell types through public single-cell transcriptomic data^23,24^, a length-associated transcriptome with a relative fold reduction of long transcripts in alveolar macrophages and alveolar type 2 cells (Supplementary Fig. 17).

The experiments were not and could not have been randomized.

#### Estimation of confidence intervals for bar plots

Bar plots represent empirically observed fractional data; for example, phenomenon present in 14 of 20 tissues would be 70%. Confidence intervals were estimated by bootstrapping 1,000 times. Each bootstrap corresponds to drawing with replacement. In the above example, this would mean doing 20 independent randomizations where for each randomization there was a 70% chance that the phenomenon would be present (and 30% chance that not).

#### Animal keeping

All mouse procedures were approved by the Institutional Animal Care and Use Committee at Northwestern University. All strains including wild-type mice were bred and housed at a specific-pathogen-free barrier facility at the Center for Comparative Medicine at Northwestern University. Male C57BL/6J mice were provided by the NIA, one of the National Institutes of Health (NIH), and were housed at Northwestern University Feinberg School of Medicine Center for Comparative Medicine for 4 weeks before euthanasia. Our rationale was to focus on a standardized murine model that is commonly used across different laboratories so that our findings could be investigated or continued by others.

Mice were euthanized by pentobarbital sodium overdose. Immediately the chest cavity was opened, and animals were perfused via the left ventricle with 10 ml of HBSS (Ca/Mn^+^). The following tissues were collected: lung, heart, liver, kidney, adrenal gland, white (perigonadal) and brown adipose tissue, skin, muscle satellite cells, frontal cortex, cerebellum, esophagus, stomach, and small and large intestines. Gut epithelial cells were isolated after flushing large intestine with EDTA/EGTA solution. Lungs were subjected to enzymatic digestion to release stromal and immune cells and sorted by FACS as described elsewhere^73^. All tissues and cells were immediately frozen on dry ice and stored at −80 °C for processing. Muscle satellite cells were prepared as described in work by Runyan et al.^74^.

#### RNA isolation and RNA sequencing

RNA was isolated using an RNeasy DNA/RNA kit after homogenization and lysis in guanidine thiocyanate buffer supplemented with β-mercaptoethanol. RNA concentration and quality were assessed using an Agilent TapeStation. RNA-seq libraries were prepared using an NEB Next RNA Ultra kit with a polyA enrichment module using an Agilent Bravo NGS Automated fluidics handling platform as described elsewhere^73^. Libraries were multiplexed and sequenced on an Illumina NextSeq 500 platform using 75 cycles of high-output flow cells and a dual indexing strategy. Our rationale was to use a protocol that had been standardized and applied by our sequencing facility.

While targeting 6 mice per age and organ, we ultimately only obtained sequenced samples for an average of 5.76 mice per age and organ (because of errors in sample isolation and/or liquid handling).

#### Bioinformatics

Sequencing reads were analyzed using an implementation of Ceto (https://github.com/ebartom/NGSbartom/) in Nextflow^75^. Briefly, BCL files were demultiplexed and converted to fastq files using bcl2fastq (version 2.17.1.14), with default parameters. The raw reads were trimmed using trimmomatic^76^ (version 0.36), with the following parameters: trailing = 30 and minlen = 20. Trimmed reads were aligned to the mouse reference genome (GRCm38.p3) with annotations from Ensembl v78 using tophat (version 2.1.0)^77^, with the following parameters: no novel junctions, read-mismatches = 2, read-edit-distance = 2 and max-multihits = 5. Aligned reads were counted using Htseq-count from htseq^78^, with the following parameters: intersection-nonempty, reverse strand, feature-type = exons, and id-attribute = gene_id. Our rationale was to use a bioinformatic setup that had been standardized and applied by our facilities.

#### Differential expression of bulk RNA sequencing

For measurements derived from multiple individuals, differential gene expression analysis was performed with DESeq2 (ref. ^69^), version 1.20 (mouse) and 1.22 (human) using an *α* value of 0.05 for the adjusted *P*-value cutoff. We subsequently only kept genes that mapped unambiguously between Ensembl gene identifiers and NCBI (Entrez) gene identifiers^13^.

To estimate the differential gene expression between individuals, we directly computed the log_2_ ratio of raw counts for transcripts detected in both individuals.

#### Characteristics of genes

For transcription factors, we mapped the Gene Transcription Regulatory Database (v18_06)^14^ to ±200 nucleotides to transcriptional start sites supplied by BioMart for the human reference genome build GRCh38.p12 and the mouse reference genome build GrCm38.p6. For miRNAs, we used miRDB (v5.0)^15^. For mature transcripts, length parameters and GC content were identified from GenBank and mapped to genes as described elsewhere using the median across different transcripts^13^. Number of exons, and their minimal, median and maximal lengths, were extracted from BioMart. For genes and chromosomes, characteristics were extracted as described elsewhere^13^. Our rationale was to consider a broad set of information that might inform on the formation or turnover of transcripts.

#### Modeling

Gradient-boosting regression models were built in scikit-learn (version 0.20.3)^16^. Of the transcripts, 90% were included as the training set and 10% were used as a test set. The 10% of the test set transcripts that had been withheld during training were used to evaluate the performance of the models. Our rationale for the gradient boosting was to account for possible non-linearities. We only considered protein-coding genes with at least one research publication and an official gene symbol, and which unambiguously mapped in a 1:1 relation between NCBI (Entrez) gene identifiers and Ensembl gene identifiers.

#### Kernel-density visualizations

Kernel-density visualizations were created with Seaborn^68^ using default parameters.

#### Comparison to two cohorts of mice

To quantitatively evaluate the performance of our machine learning approach, we first estimated the maximal performance that should be achievable by our experimental data. The latter will depend on biological, experimental and technical variability, the true number of genes that change expression during aging and their true magnitudes of change, and the sensitivity of RNA-seq to detect transcript molecules and their change. We built upon the two-cohort design of our experimental survey and compared transcriptional fold changes between the two cohorts. Specifically, the six mice of each age and tissue pair had drawn from two cohorts with three mice per age each that were euthanized on different days (or two and three mice per age if we only obtained samples from five mice, or two mice per age if we only obtained samples from four mice). As anticipated^2^, Spearman correlations for the relative fold changes between measurements obtained by two cohorts of mice appeared small (interquartile range (0, 0.250)) and, in some cases, even slightly negative (Supplementary Fig. 2b). Of direct relevance to our efforts to evaluate the performance of our machine learning approach, the Spearman correlations between observed relative fold changes and predicted relative fold changes (Extended Data Fig. 1a), however, resembled or exceeded those observed between both cohorts (Supplementary Fig. 2b).

#### Comparison to differential gene expression analysis

We found good agreement between the prediction accuracy of our gradient-boosting regression models and the number of expressed genes detected to be differentially expressed (Extended Data Fig. 2). We also found that gradient-boosting regression reached statistical significance (*P* < 0.01) for tissue–age pairs where the transcript of no single gene was statistically significantly differentially expressed at a Benjamini–Hochberg-adjusted *P*-value cutoff of 0.05 (Extended Data Fig. 2c), suggesting that differential gene expression analyses might yield false-negative findings.

#### Alternate bioinformatics

To ensure robustness of results beyond individual bioinformatic pipelines, we reanalyzed in-house bulk RNA-seq datasets using the publicly available nf-core/RNA-seq pipeline version 1.4.2 implemented in Nextflow 19.10.0 using Singularity 3.2.1–1 with the minimal command: nextflow run nf-core/rnaseq -r 1.4.2 –singleEnd -profile singularity –unStranded –fc_group_features_type ‘gene_id’^75,79,80^. Briefly, lane-level reads were trimmed using trimGalore! 0.6.4 and aligned to the GRCm38 genome using STAR 2.6.1d^81^. Gene-level assignment was then performed using featureCounts (1.6.4)^82^. Included in the nf-core/RNA-seq QC output is a matrix of Pearson correlations of log_2_(CPM) values generated using edgeR (3.26.5)^83^. Lanes with Pearson *R* < 0.7 compared to all other lanes constituting a given sample were excluded from further analysis. Extant lanes were then merged by sample with SAMtools 1.6 using the minimal command ‘samtools merge --r’^84^. Merged BAM files were then reassigned using Rsubread 1.32.4 in R 3.5.1 using the minimal command ‘featureCounts(files, annot.inbuilt = ‘mm10’, minFragLength = 25)’ and merged into separate datasets by tissue in DESeq2 (1.22.2)^69,82^. Using a combined factor of age, and influenza dose (plaque-forming units), differential expression analysis (DEA) was performed with the formula ‘~combined’. A local estimate of gene dispersion best fit observed dispersions in all cases. DEA was therefore performed using the minimal command ‘DESeq(dataset, fitType = ‘local’, parallel = T)’. DEA tables were output for all permutations of age and influenza dose for a given tissue and analyzed as above.

#### Length correlations

To avoid assumptions on linearity, we used the Spearman correlation between transcript length and relative fold change of transcripts in older individuals over younger ones. Significance was obtained through the scipy.stats (version 1.2.1) implementation of the Spearman correlation^67^.

For human GTEx data, we restricted our analysis to tissues present in samples from young and middle-aged and old donors.

#### Quantification through difference in median transcript length

We alternatively quantified the length-associated transcriptome imbalance through the difference in median transcript length among those genes that, in a differential gene expression analysis, showed a significant relative fold increase and the transcript length of those genes that showed significant relative fold decrease.

#### Technical robustness checks

As technical artifacts could affect transcripts according to length^40,42,43^, we further tested the robustness of the recovered length-associated transcriptome imbalance.

First, we repeated our initial analysis (Fig. 1c) and filtered the datasets for those organs where the relative fold changes correlated across both cohorts exceed a Spearman correlation of 0.2 and found that the correlation between transcript lengths and relative fold changes persisted (Supplementary Fig. 6a). Further, and based on counting the tissues exceeding a Spearman correlation of 0.2, we noted, as expected^21^, that the age-dependent changes were most reproducible when comparing 24-month-old organs against 4-month-old organs (Supplementary Fig. 6a). Second, we did not observe changes in RNA integrity with age (Supplementary Fig. 6b). Third, we observed the same correlations between transcript length and relative fold change if we removed the requirement for the annotation of genes to be supported by several lines of evidence such as gene symbols or literature published in MEDLINE, lowering the likelihood that our findings could be an artifact of the stringency of applied gene annotations (Supplementary Fig. 6c).

For our fourth robustness check, we excluded samples where the sequencing data yielded lower-quality metrics. Reassuringly, this yielded practically identical measurements of the length correlations—with two conditions (12-month-old lung, and 18-month-old large intestine) even turning a positive correlation (favoring long genes) toward the more negative correlation (disfavoring long genes) as seen for the majority of conditions (Supplementary Fig. 6d). Solely, the condition with the strongest imbalance (18-month-old large intestine), now showed a weaker imbalance (Spearman correlation with length, from −0.67 to −0.24; Supplementary Fig. 6d). As a fifth robustness check, and after our initial discovery of the age-dependent length correlation, we asked one team member, who was not involved in the design or execution of the initial bioinformatic preprocessing, to prepare an independent bioinformatic pipeline, QC and sample filtering, and differential gene expression analysis. To mitigate the potential risk associated with custom pipelines, the team member used nf-core/RNA-seq, which provides community-curated bioinformatics pipelines^80^. Again, we observed practically identical measurements of the length correlations—with 18-month-old hearts again turning a positive correlation toward the more representative negative correlation (Supplementary Fig. 6e). Sixth, we excluded, after the bioinformatic processing and differential gene expression analysis, genes with a known annotation relating to the inflammatory response (genes that tend to be short) and neuronal genes (genes that tend to be long). Again, we observed practically identical measurements of the length correlations (Supplementary Fig. 10f).

Seventh, after a non-parametric LOWESS regression between transcript length and relative fold changes^42,85^, we no longer observed any length-associated transcriptome imbalance when considering the correlation between transcript lengths and residual fold changes after LOWESS regression. This suggests that the length-associated transcriptome imbalance is a transcriptome-wide phenomenon that could be accounted for by transcript length alone (Supplementary Fig. 10g).

Eighth, reanalyzing published transcript degradation rates^43^, we found that longer transcripts were slightly more stable when compared to other transcripts across the genome (Spearman, −0.03; Supplementary Fig. 10h).

Ninth, we tested if a length-associated transcriptome imbalance persists if, instead of transcript length, we considered other, correlated (Extended Data Fig. 3a) readouts of length. We therefore measured the correlation between the observed relative fold changes and the median gene length (Supplementary Fig. 7a,b) and the median length of the coding sequence (Supplementary Fig. 7c,d). Indeed, we continued to detect a length-associated transcriptome imbalance (Supplementary Fig. 7).

#### Biological variation

We determined whether our sample size of six mice would be sufficient to conclude whether the length-associated transcriptome imbalance measured between mice of distinct chronological ages would exceed the length-associated transcriptome imbalance seen among mice of the same chronological age. We performed retrospective subsampling of all mice of a given chronological age and separated them into two equally sized groups (or three versus two mice if one sample had not been processed; Supplementary Table 1). For each possible permutation of separating mice into two different groups, we measured the correlation between transcript lengths and the relative fold changes of transcripts between those two groups. Comparing these permutations against the length-associated imbalance that we observed across distinct chronological ages, we inferred that—given the number of 17 organs and target sample size of 6 mice—we can presently only conclude for the comparison between 4-month-old and 24-month-old mice that the majority of organs demonstrate an imbalance with age that exceeds interindividual variability (Mann–Whitney *U* < 0.001; Fig. 1c and Supplementary Fig. 8a,b).

Next, we performed all pairwise comparisons between all mice of a given age relative to all 4-month-old mice. Reminiscent of our preceding analysis, we observed the imbalance was most pronounced when comparing 24-month-old mice against 4-month-old mice (Supplementary Fig. 8b). For all but two organs (lung and skin; Supplementary Fig. 8c), we found a relative fold decrease of long transcripts for more than half of all pairwise comparisons. Notably, the occurrence of a general trend of the length correlation by age does not indicate that all possible combinations of mice show a fitting reduction of long transcripts by age. Across all combinations, and organs, we found this fraction to be 67% (interquartile range (53%, 81%)), indicating that the length-dependent imbalance against long transcripts is not fully penetrant (Extended Data Fig. 8c).

#### NanoString

NanoString analysis was performed by NUSeq Core using the metabolism panel (v1).

Samples were hybridized overnight at 65 °C for 16 h, according to NanoString’s recommended protocol. As all samples were DV300 >90% (that is, 90% of the RNA species should be of 300 nucleotides or longer), 75 ng input RNA was used for each hybridization reaction. Samples were then immediately loaded into the NanoString nCounter cartridges and processed. Differential expression was performed using nSolver version 4.0.70 (NanoString) between the 4-month group and the 24-month group using default parameters.

#### Reanalysis of previous studies

We considered genes reported to show a relative fold decrease or relative fold increase in earlier studies. For mice and rats, we used protein-coding genes with at least one research publication and an official gene symbol, and the median transcript lengths derived from GenBank. For killifish, we used genes and gene lengths as reported by Reichwald et al.^19^.

For studies reporting transcriptome measurements (Schaum et al.^22^ and Shavlakadze et al.^21^), we used a significance threshold of *P* < 0.01 for the correlation between transcript length and relative fold changes. For studies that only provided lists of differentially expressed genes and their relative fold increase or relative fold decrease (Benayoun et al.^20^ and Reichwald et al.^19^), we applied a two-sided Mann–Whitney *U* test, to determine whether the median transcript length of transcripts with a relative fold increase was different at the *P* < 0.01 level from the median length of transcripts with a relative fold decrease. In case that multiple ages were tested separately by individual studies, we selected the ages closest to 4 and 24 months of age for young and old, respectively.

#### Single-cell transcriptome imbalance

As cell types, we considered the cell_type and cell_ontology_class columns within the respective meta-data tables contained in the h5ad files of Kimmel et al.^23^ and Tabula Muris Senis^24^. We only considered protein-coding genes that were detected in at least one cell of a given cell type in an individual organ in a given study. We determined the transcriptome imbalance for each cell type by correlating transcript length against the log_2_-fold ratio formed by the summed raw counts of the older animals divided by the summed raw counts of the younger animals.

#### Exclusion of genes with inflammation and neuronal function

To determine robustness beyond short stress-induced genes and long neuronal genes, we removed them from our analysis after bioinformatic processing. We excluded genes if the lowercase spelling of the Gene Ontology term contained ‘immune’, ‘stress’, ‘inflamm’ or ‘infect’, ‘brain’, ‘neuro’, ‘nerv’, ‘cerebral’, ‘cortex’ or ‘memory’.

#### Mapping of rhesus macaque genes

For the analysis of Murray et al.^86^, we mapped genes to human transcript length through gene symbols shared with humans.

#### Analysis of Flynn et al.

Contrasting other anti-interventions, we reanalyzed the raw data of Flynn et al.^87^ as, despite the statement in a corresponding figure legend, their study did not include the corresponding supplementary table with differential gene expression results (Supplementary Table 3).

#### Functional enrichments

We considered the genes with the 5% shortest and 5% longest median transcript length. We used the annotation of pro-longevity and anti-longevity genes from HAGR^29,88,89^. If genes were simultaneously annotated as pro-longevity and anti-longevity genes (21 of 665 in human, 19 of 665 in mice), we kept both annotations. After intersecting with protein-coding genes with a reported transcript length, this yielded 417 anti-longevity and 267 pro-longevity genes in humans, and 307 anti-longevity and 200 pro-longevity genes in mice. Our rationale was to adhere to the most comprehensive curation of individual longevity genes.

For differential enrichment, we considered genes enriched among the genes with transcripts of one length extreme (5% shortest and 5% longest) at a Benjamini–Hochberg *P* value < 0.05 and depleted among the genes with the other length extreme. Unless indicated otherwise, we restricted the background gene lists for the enrichment to those genes carrying at least one annotation.

#### False-positive rate of opposing enrichment and depletion

To understand whether an opposing enrichment and depletion of Gene Ontology terms is common, we asked explicitly whether the number of categories that were opposingly enriched among short and long transcripts (Supplementary Tables 7–10) was higher than would be expected by chance when comparing two random samples drawn from the length distributions observed in the mouse and human genomes. We performed 100 randomizations for mouse and 100 randomizations for human genes. For mice, no single randomization identified any annotation. For humans, 1 of 100 randomizations identified a single annotation (of 14,223 possible annotations for mouse genes, and 15,371 possible annotations for human genes), while the remaining 99 randomizations identified no annotations. These values were significantly lower than the number of annotations that we observed when using the shortest and longest transcripts of mouse and humans, that is, 138 and 140, respectively (Supplementary Tables 7–10).

#### Alternative enrichment analysis for longevity genes

As an additional analysis, which we expected to have lower statistical power due to the increased number of uninformative genes, we repeated our enrichment analysis while considering all protein-coding genes, rather than solely those occurring in the database used for annotation (Extended Data Fig. 9g,h). We observed that the directionality in these trends with aging always persisted in human and persisted in three of four cases in mice—with the exception being anti-longevity genes among the longest transcripts. Notably, for humans, one case—the depletion of anti-longevity genes from the longest transcripts did not reach statistical significance (two-sided Fisher’s exact *P* = 0.31). Further, in mice, the enrichment of anti-longevity genes among the shortest transcripts did not reach statistical significance (two-sided Fisher’s exact *P* = 0.31).

#### Annotation network construction

To organize annotations according to their similarity in the shared genes rather than the human-imposed hierarchical organization, we represented the annotations found to be enriched as nodes, drew edges between two nodes if at least one gene carried both annotations and simply the network as follows: Starting with node with the fewest attached genes, we kept the edge from that node to the node with the largest intersection set of attached genes. In case of a tie, that is, in case there were several nodes with intersection sets of attached genes of the same size, we kept the edge to the node with the fewest number of attached genes. In case a tie remained, we kept the edge to the annotation node with the fewest genes attached but now including genes that were not included in the enrichment analysis. We repeated this procedure for the other nodes in order of increasing number of genes attached.

### Reporting summary

Further information on research design is available in the Nature Portfolio Reporting Summary linked to this article.

## Supplementary information

Supplementary Information Supplementary Figs. 1–17

Reporting Summary

Supplementary Tables 1–10 Supplementary Table 1. Number of mice analyzed for each combination of tissue and age, related to Fig. 1. Supplementary Table 2. Median rank of distinct features for different tissues at distinct ages compared to 4-month-old animals. Columns named by tissue_age, related to Fig. 1. Supplementary Table 3. Correlations between transcript lengths and relative fold changes in cell types identified in single-cell studies, related to Fig. 2. Supplementary Table 4. Median rank of distinct features for different tissues at distinct ages compared to young human donors (20–39 years). Columns named by gender_tissue_old decade_young_decade, related to Fig. 3. Supplementary Table 5. Transcriptome imbalance in female samples of the GTEx. Column ‘comparison’ shows compared decades. ‘rho’ represents the correlation between transcript lengths and relative fold changes, and ‘pval’ indicates the corresponding significance, related to Fig. 3. Supplementary Table 6. Transcriptome imbalance in male samples of the GTEx. Column comparison shows compared decades. rho is the correlation between transcript lengths and relative fold changes, and pval is the corresponding significance, related to Fig. 3. Supplementary Table 7. Gene Ontologies enriched among mouse genes with 5% shortest median transcript length, related to Fig. 5. Supplementary Table 8. Gene Ontologies enriched among mouse genes with 5% longest median transcript length, related to Fig. 5. Supplementary Table 9. Gene Ontologies enriched among human genes with 5% shortest median transcript length, related to Fig. 5. Supplementary Table 10. Gene Ontologies enriched among human genes with 5% longest median transcript length, related to Fig. 5.

Supplementary Data 1 Supplementary Network 1. Gene Ontology network of genes with opposing functional enrichments among shortest and longest transcripts, related to Fig. 5.

## Data Availability

RNA-seq data created during this study, and used for Fig. 1 have been deposited under GSE141252. Data underlying other figures have been generated by other research groups and are available from them and/or their respective publications. Externally generated data can be obtained from the following resources listed according to their order of usage: Gallego Romero et al.^43^ (Additional File 11), Schaum et al.^22^ (https://figshare.com/articles/Differential_Gene_Expression/12227531), Benayoun et al.^20^ (Supplementary Table 4), Shavlakadze et al.^21^ (Supplementary Table 1), Reichwald et al.^19^ (Supplementary Data 4), Kimmel et al.^23^ (http://mca.research.calicolabs.com), Tabula Muris Senis (https://figshare.com/articles/Processed_files_to_use_with_scanpy_/8273102 and https://figshare.com/articles/Processed_files_to_use_with_scanpy_/8273102), GTEx (https://gtexportal.org/home/datasets version 7; dbGaP accession phs000424.v7); Martinez-Nunez et al.^91^ (Supplementary Data file); Flynn et al.^87^ (GSE48043); Mattson et al.^92^ (Table S2); Amador-Noguez et al.^90^ (Supplementary Table 1 for Ames Dwarf mice and Supplementary Table 2 for Little mice), Ng et al.^95^ (Table S8), Murray et al.^86^ (Supplementary Tables 1A and 1B); Luizon et al.^96^ (Supplementary Table 1); Hofmann et al.^97^ (Supplementary Fig. 11); Dembic et al.^98^ (Appendix); Selman et al.^94^ (Supplementary Tables 2–4); Hoffman et al.^93^ (Supplementary Table 5); Jochems et al.^99^ (Supplementary Table 2); Lu et al.^28^ (Supplementary Fig. 4). Additional data used in this study were: Gene Transcription Regulation Database version 18.06 (http://gtrd.biouml.org:8888/downloads/18.06/); miRDB version 5.0 (http://mirdb.org/download/miRDB_v5.0_prediction_result.txt.gz); Genes and transcript sequences from GenBank (GRCh38.p10 for human, and GRCm38.p5 for mice; ftp://ftp.ncbi.nlm.nih.gov/genomes); GTEx Portal version 7 (https://www.gtexportal.org/home/datasets/); Exons from Biomart, using human genome GRCh38.p12 and mouse genome GRCm38.p6 (https://www.ensembl.org/biomart/); HAGR^29,88,89^, specifically Longevity Map Build 3 and GenAge Build 19 (https://genomics.senescence.info/); Homologene, version 68 (https://ftp.ncbi.nlm.nih.gov/pub/HomoloGene/). Gene Ontologies using the mapping to NCBI were provided by the National Library of Medicine (https://ftp.ncbi.nlm.nih.gov/gene/DATA/gene2go.gz)

## Code availability

Code used during data analysis can be accessed through https://github.com/NUPulmonary/stoeger_et_2022_transcriptome_imbalance/.
