# Supplementary material for: Aging is associated with a systemic length-associated transcriptome imbalance
Source: Nat Aging. 2022 Dec 9;2(12):1191–206. doi: 10.1038/s43587-022-00317-6 (PMC10154227; doi:10.1038/s43587-022-00317-6)
Supplement: Supplementary file 1 — Supplementary Figs. 1–17 [file 43587_2022_317_MOESM1_ESM.pdf]

nature aging

Analysis

https://doi.org/10.1038/s43587-022-00317-6

Aging is associated with a systemic length-associated transcriptome imbalance

In the format provided by the authors and unedited

Supplementary Fig. 1

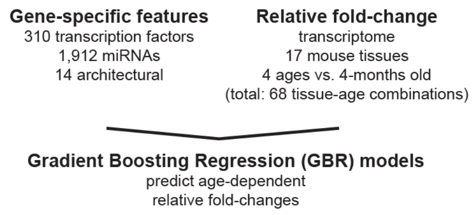

Supplementary Fig. 1. Overview of approach, related to Fig. 1.

For every tissue-age pair, we train a gradient boosting regression (GBR) model to predict the relative fold-change in regard to 4-month-old mice of transcripts. Our dependent variables are the relative fold-change in transcript abundance of 9-, 12-, 18- or 24-months old mice against data from 4-months old mice for each of 17 tissues. The models consider 2,236 features that capture information about known gene-specific regulators (transcription factors, miRNAs) and structural characteristics of genes and transcripts.

Supplementary Fig. 2

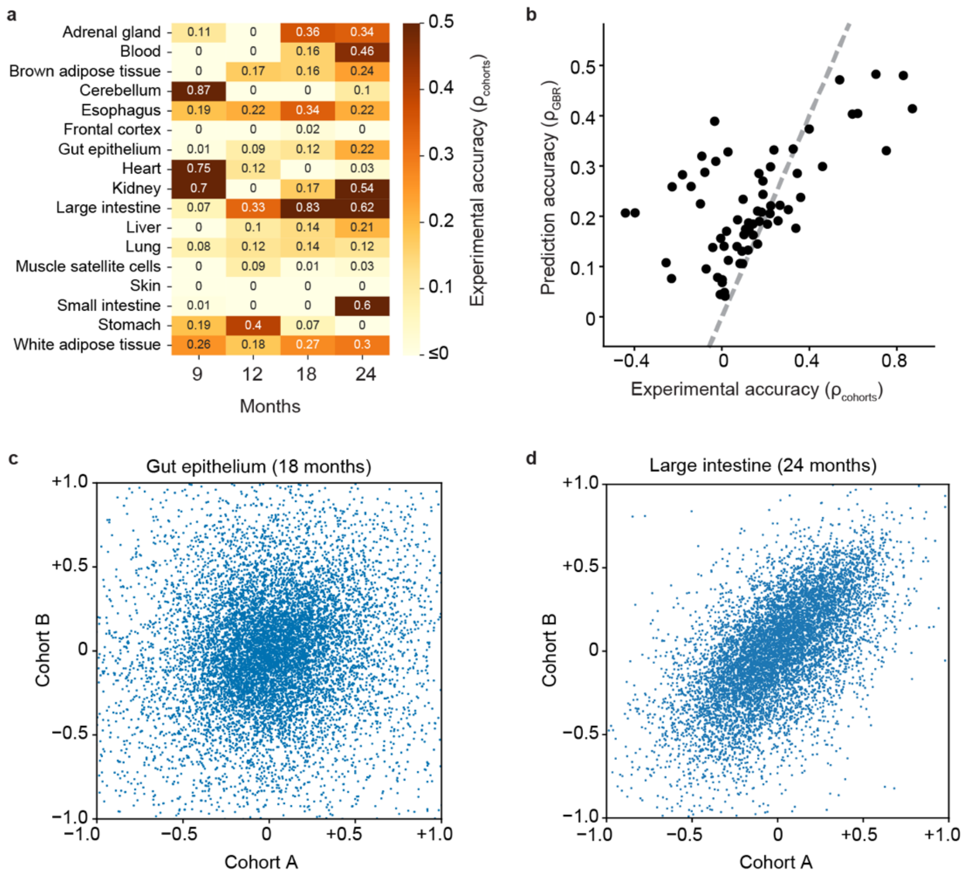

Supplementary Fig. 2. Correlation between two cohorts with 3 mice each, related to Fig. 1. (**a**) We defined experimental accuracy ($\rho_{cohorts}$) as the Spearman correlation between the relative fold-changes observed across two cohorts, which each consist of 3 mice sacrificed on different days. For consistency of the color-scale across different figures correlations below 0 are set to 0. (**b**) Comparison of prediction accuracy of GBR model (ρ_{GBR}) versus the empirically observed experimental accuracy formed correlation between the relative fold-changes between the two cohorts ($\rho_{cohorts}$). Dashed grey line indicates identity (x = y). Reassuringly, we find a positive correlation between the two measures indicating that the ability to predict relative fold-changes increases with the strength of the correlations between the two cohorts. (**c**) To illustrate the range of change in strength of the correlations, we show for individual genes (blue dots) relative fold-changes of their transcripts as experimentally observed between both cohorts, of 11,263 genes with transcripts detected in both cohorts 18-month-old gut epithelium. (**d**) As panel **c**, but for 8,239 genes with transcripts detected in both cohorts 24-month-old large intestine.

Supplementary Fig. 3

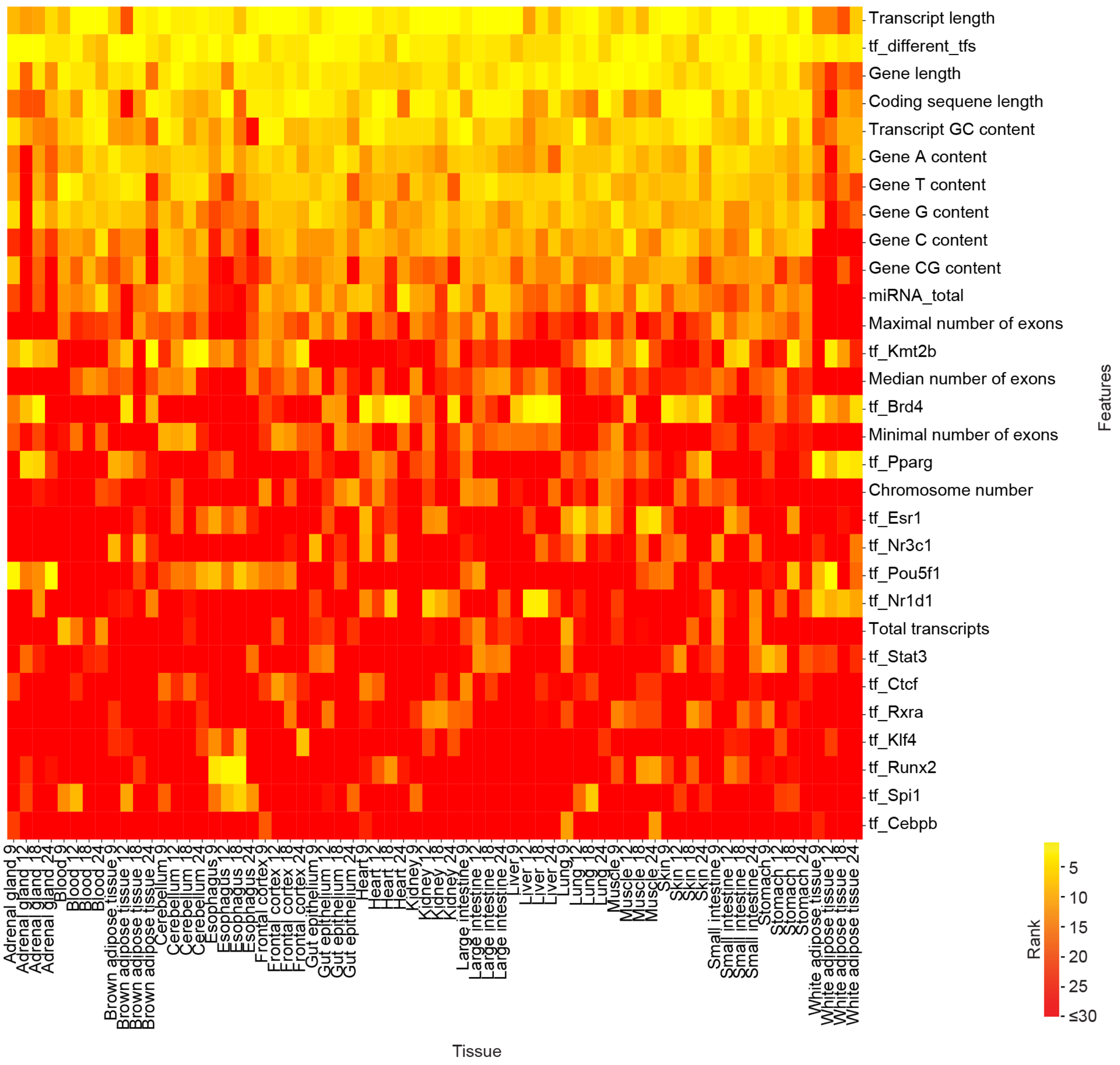

Supplementary Fig. 3. Top 30 most informative features ordered by median rank for every tissue-age pair, related to Fig. 1.

Supplementary Fig. 4

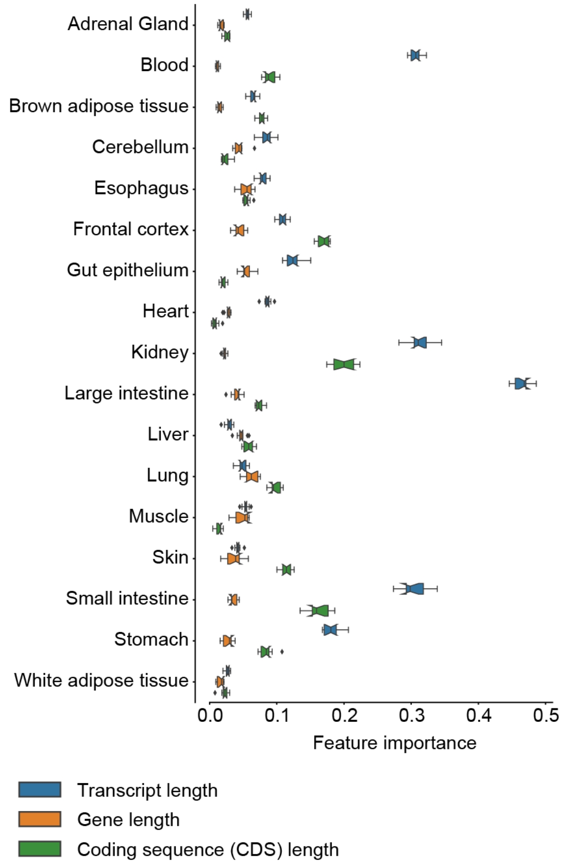

Supplementary Fig. 4. Feature importance of length-related features for tissues collected from 24-months old mice, related to Fig. 1.

Feature importance as inferred by contribution to gradient boosting regression models across all 17 tissues. Boxplots show n=10 bootstraps, with each using a random sample corresponding to 90% of the protein-coding genes. In boxplots center is median, notches bootstrapped 95% confidence interval of median, bounds of box 25% and 75% percentiles, whiskers extend height of box 1.5 times, minima and maxima observed minima and maxima.

Supplementary Fig. 5

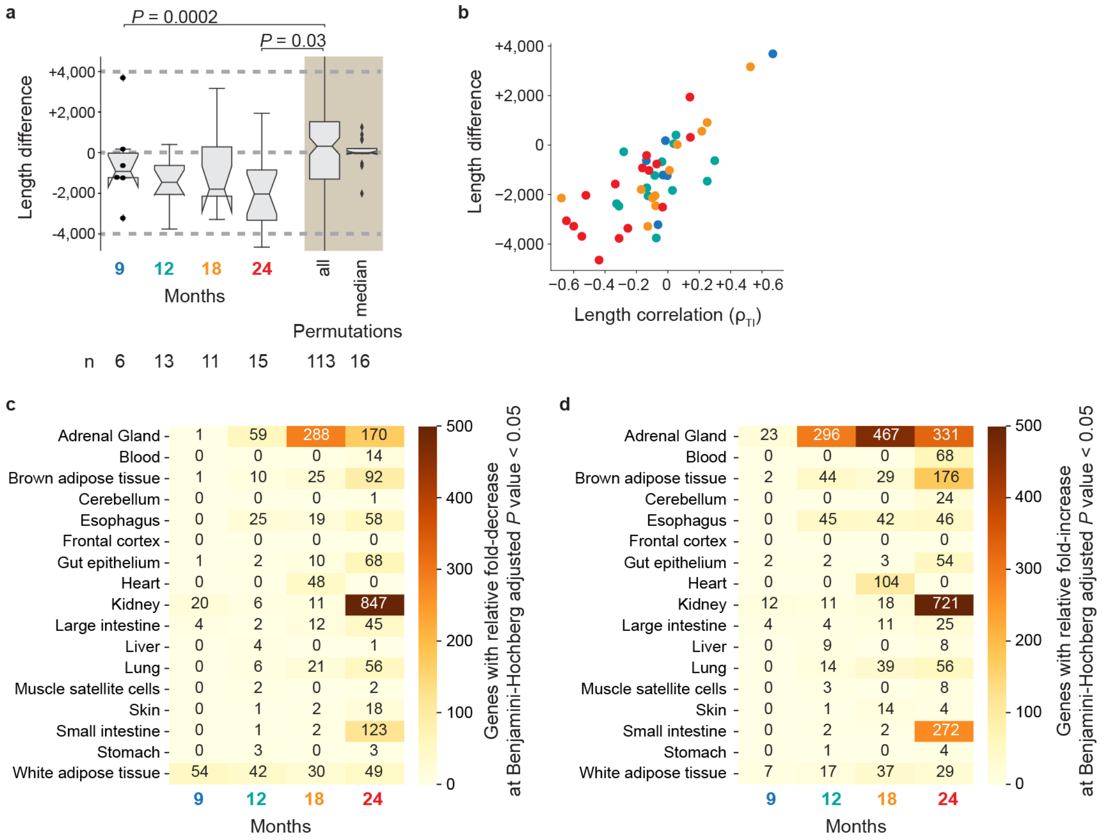

Supplementary Fig. 5. Transcriptome imbalance can alternatively be quantified through differences in the median transcript length of genes with significant relative fold-increases versus significant relative fold-decreases, related to Fig. 1.

(**a**) As Fig. 1c but showing the length difference in median transcript length of genes with significant (adjusted *P* value < 0.05) relative fold-increase minus the median transcript length of genes with a significant (adjusted *P* value < 0.05) negative relative fold-change. Under “all,” we show all such permutations from data across all 17 tissues. Permutations separated mice into 3 vs. 3 groups (or 3 vs. 2 groups if not all samples had been processed successfully) and then asked for the length association in the resulting relative fold-changes between those groups. We performed all permutations possible among all mice. Under “median,” we show the median length-associated changes for each tissue across all possible permutations. n= number of tissues and/or permutations. Notably, only tissues with at least one gene with positive and relative fold-decrease are considered. In boxplots center is median, notches bootstrapped 95% confidence interval of median, bounds of box 25% and 75% percentiles, whiskers extend height of box 1.5 times, minima and maxima observed minima and maxima except for ‘all’ which was cropped to allowed for bigger display of empirically observed range. Black dots indicate individual tissues for conditions where number of tissues < 10. (**b**) Comparison between length correlation (ρ_{TI}) and length difference for individual tissues. Colors indicate ages as in panel **a**.

Note the strong correlation between the outcomes of the two approaches. (**c**) Number of genes with a significant relative fold-decrease at an adjusted *P* value < 0.05. *P* value has been obtained by DESeq2 which uses two-sided Wald test. (**d**) Number of genes with a significant relative fold-increase at an adjusted *P* value < 0.05. *P* value has been obtained by DESeq2 which uses two-sided Wald test. Be aware that the sum of values in panels **c** and **d** is marginally lower than in Extended Data Fig. 2 as not all mouse protein-coding genes are annotated with transcripts length in our source data base, which only included verified transcripts.

Supplementary Fig. 6

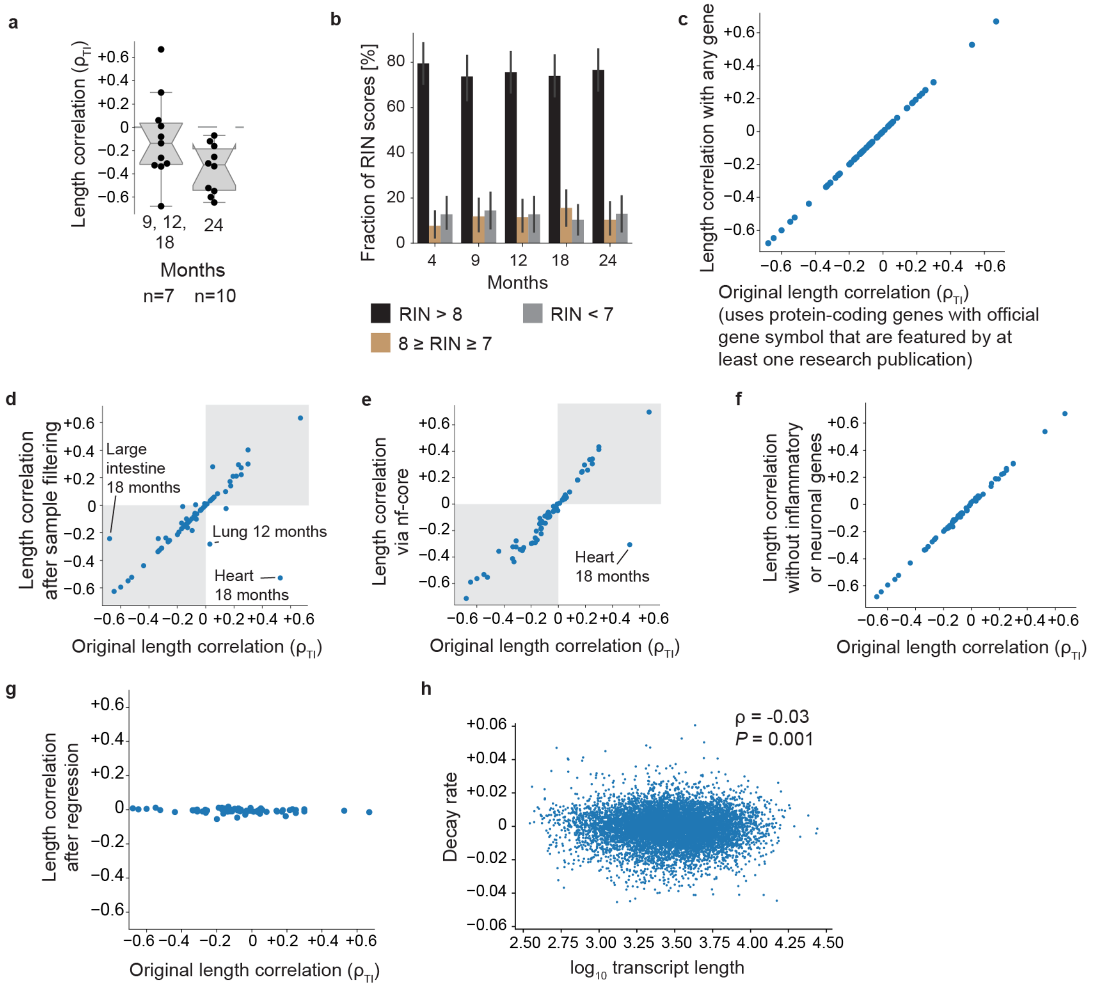

Supplementary Fig. 6. Additional control analyses on correlation between transcript lengths and relative fold-changes, related to Fig. 1.

(**a**) Length correlations as reported in Fig. 1c but filtering for n=tissues with an empirically observed experimental accuracy above 0.2 between the two cohorts sacrificed on different days ($\rho_{cohorts}$) as in Supplementary Fig. 2b. In boxplots center is median, notches bootstrapped 95% confidence interval of median, bounds of box 25% and 75% percentiles, whiskers extend height of box 1.5 times, minima and maxima observed minima and maxima. Black dots indicate individual tissues. (**b**) RNA Integrity Scores (RIN Scores) for individual samples with n=78, 76, 78, , 77 and 77 for 4, 9, 12, 18, 24 months, respectively. The center of the error bars is the empirically observed fraction of RIN scores within a given interval. Note that individual data points cannot be shown as bars represent fraction of RIN scores. Thus, error bars are 95% confidence interval of bootstrapped estimates of these fractions. (**c**) Comparison of original length correlation (ρ_{TI}) for tissues of 9-, 12-, 18-, and 24-month-old mice as calculated in Fig. 1c

against length correlation computed following exclusion all genes that are not protein-coding genes, or are not mentioned in at least one publication in MedLine, or do not carry an official gene symbol. Number of samples at a given age are given in Supplementary Table 1. Note the strong correlation between the outcomes of the two approaches. (**d**) Comparison of original length correlation (ρ_{Tl}) for tissues of 9-, 12-, 18-, and 24-month-old mice relative to 4-month-old mice as calculated in Fig. 1c against length correlation computed following exclusion of samples with comparably lower quality metrics. Note the strong correlation between the outcomes of the two approaches. Indeed, we find significant differences for only 3 cases, with 2 of those cases now also following the general tendency of a relative fold-decrease rather than relative fold-increase of long transcripts with age. (**e**) Comparison of original length correlation (ρ_{Tl}) for tissues of 9-, 12-, 18-, and 24-month-old mice as calculated in Fig. 1c against length correlations computed following independent bioinformatic pipeline from nf-core[80]. Note the strong correlation between the outcomes of the two approaches. We find significant differences for only 1 case; with this 1 case now also following the general tendency of a relative fold-decrease rather than relative fold-increase of long transcripts with age. (**f**) Comparison of original length correlation (ρ_{Tl}) for tissues of 9-, 12-, 18-, and 24-month-old mice as calculated in Fig. 1c against length correlation for tissues of 9-, 12-, 18-, and 24-month-old mice after excluding genes relating to inflammation and neuronal processes. Note the strong correlation between the outcomes of the two approaches. (**g**) Comparison of original length correlation (ρ_{Tl}) for tissues of 9-, 12-, 18-, and 24-month-old mice as calculated in Fig. 1c against ρ_{Tl} for tissues of 9-, 12-, 18-, and 24-month-old mice after LOWESS regression of fold-changes against median transcript length. (**h**) Spearman correlation between transcript lengths, and relative fold-changes reported by Gallego Romero et al. [43]. Significance is determined using t-distribution for two-sided significance test for Spearman correlation[66]

Supplementary Fig. 7

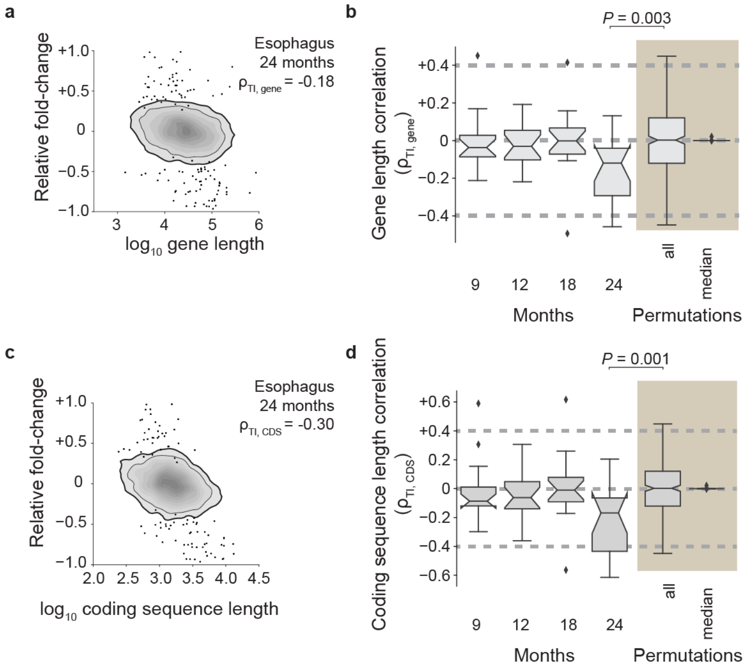

Supplementary Fig. 7. Age-dependent length correlation also persists when computed against gene-length and coding sequence (CDS) length, related to Fig. 1.

(**a**) Association between $\log_{10}$ gene length and the relative fold-change in transcript abundance between esophagus samples from 4- and 24-month-old mice. We quantify the observed length-associated transcriptome imbalances using the Spearman correlation ($\rho_{TI,\ gene}$) between $\log_{10}$ gene length and relative fold-changes. We show kernel density estimates using a linear gray scale. The thin and thicker black lines indicate outermost boundaries of 80% and 90% of kernel density estimate, respectively. We highlight with black circles those genes identified by gene-specific differential expression at an adjusted *P* value below 0.05. 67 genes show a significant relative fold-decrease and 62 a significant relative fold-increase (adjusted *P* value < 0.05). *P* values obtained by DESeq2 which uses two-sided Wald test. **(b)** Boxplot representation of the 68 $\rho_{TI,\ gene}$ obtained from age comparisons across the 17 tissues considered in our study. To provide a baseline for individual sample variability, on the right (shaded in beige), we show the boxplot of values of $\rho_{TI,gene}$ obtained from calculating relative fold-changes across all permutations of assigning same age mice to two groups. Under "all," we show all such permutations from data across all 17 tissues. Permutations separated mice into 3 vs. 3 groups (or 3 vs. 2 groups if not all samples had been processed successfully) and then asked for the length association in the resulting relative fold-changes between those groups. We performed all permutations possible among all mice. Under "median," we show the median length-associated changes for each tissue across all possible permutations. We estimated *P* values with two-sided Mann-Whitney *U* tests. In boxplots center is median, notches bootstrapped 95% confidence interval of median, bounds of box 25% and 75% percentiles, whiskers extend height

of box 1.5 times, minima and maxima observed minima and maxima. Notches suggest that the differences between 9-, 12-, and 18-month-old mice are not statistically significant. (**c**) As panel **a**, but for median length of coding sequence with 58 genes showing a significant relative fold-decrease and 46 a significant relative fold-increase (adjusted *P* value < 0.05). (**d**) As panel **b**, but for median length of coding sequence. For number of tissues and permutations see Fig. 1c.

Supplementary Fig. 8

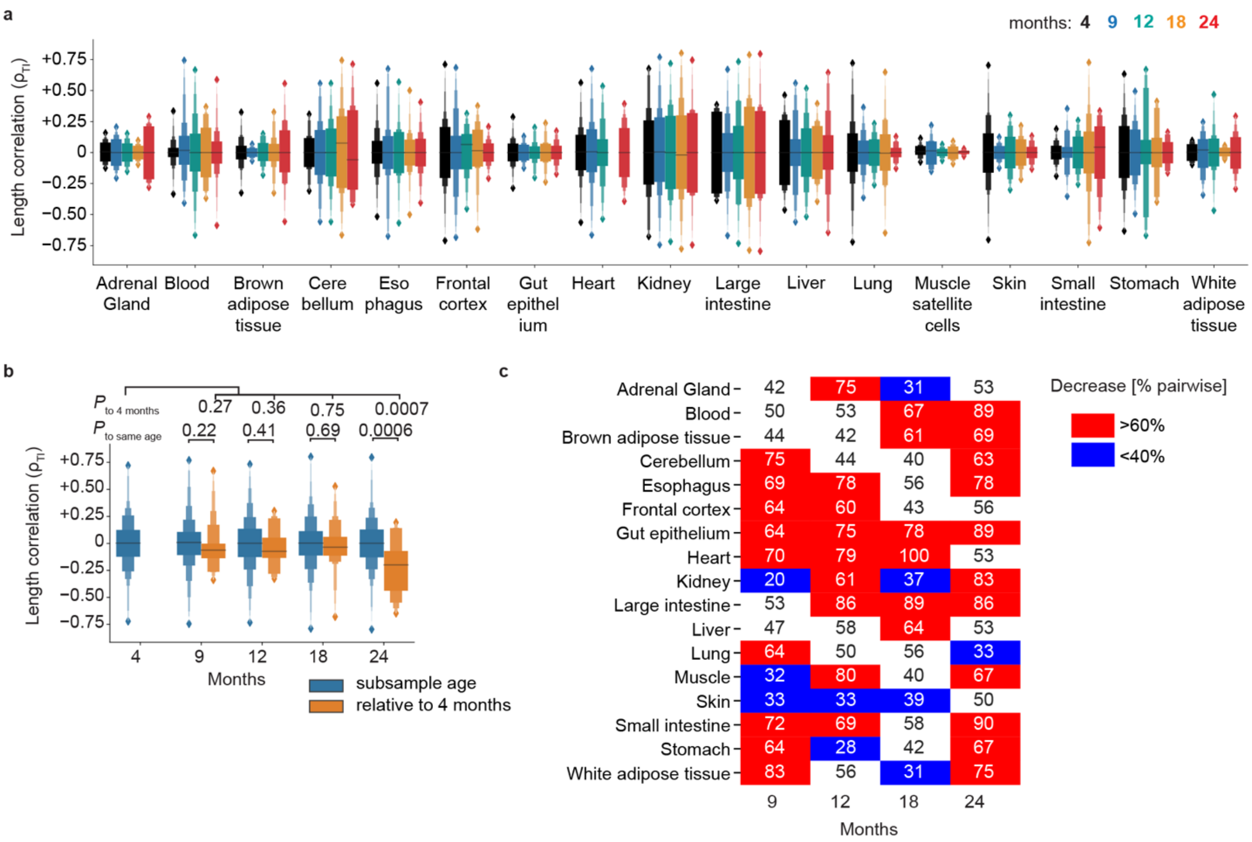

Supplementary Fig. 8. Variability across biological replicates, related to Fig. 1.
(**a**) For all samples in each age-tissue pair, we consider all distinct combinations for assigning mice to two equal-sized groups (yielding 71 age-tissue pairs where all 20 combinations are possible, and 11 pairs with 10 combinations and 2 age-tissue pairs with 6 combinations, see Supplementary Table 1). We calculate relative fold-change from one group to another. Color indicates mice ages. (**b**) Blue letter-value plots show data from panel **a** but aggregated across all tissues for each age. Orange letter-value plots show distribution of ρ_{TI} from the calculated relative fold-changes for all mice relative to 4-months-old mice (data identical to non-shaded boxplots in Fig. 1c). $P_{\text{to 4 months}}$ is two-sided Mann-Whitney U relative to sub-samples groups of 4-month-old animals, and $P_{\text{to same age}}$ is two-sided Mann-Whitney U relative to sub-samples of groups animals of the same age. (**c**) Percentage of all pairwise comparisons between individuals of different ages that display an anticorrelation between transcript length and age-depend relative fold-changes relative to 4-month-old mice, that is, $\rho_{TI} < 0$. (**a, b**) Letter-value plots shows median as black lines, boxes of decreasing width with outer edge corresponding iteratively to percentiles of data according to letter ratios (+/- 25%, +/-37.5, +/-43.75, etc.), and as diamonds the minima and maxima, which are observed minima and maxima.

Supplementary Fig. 9

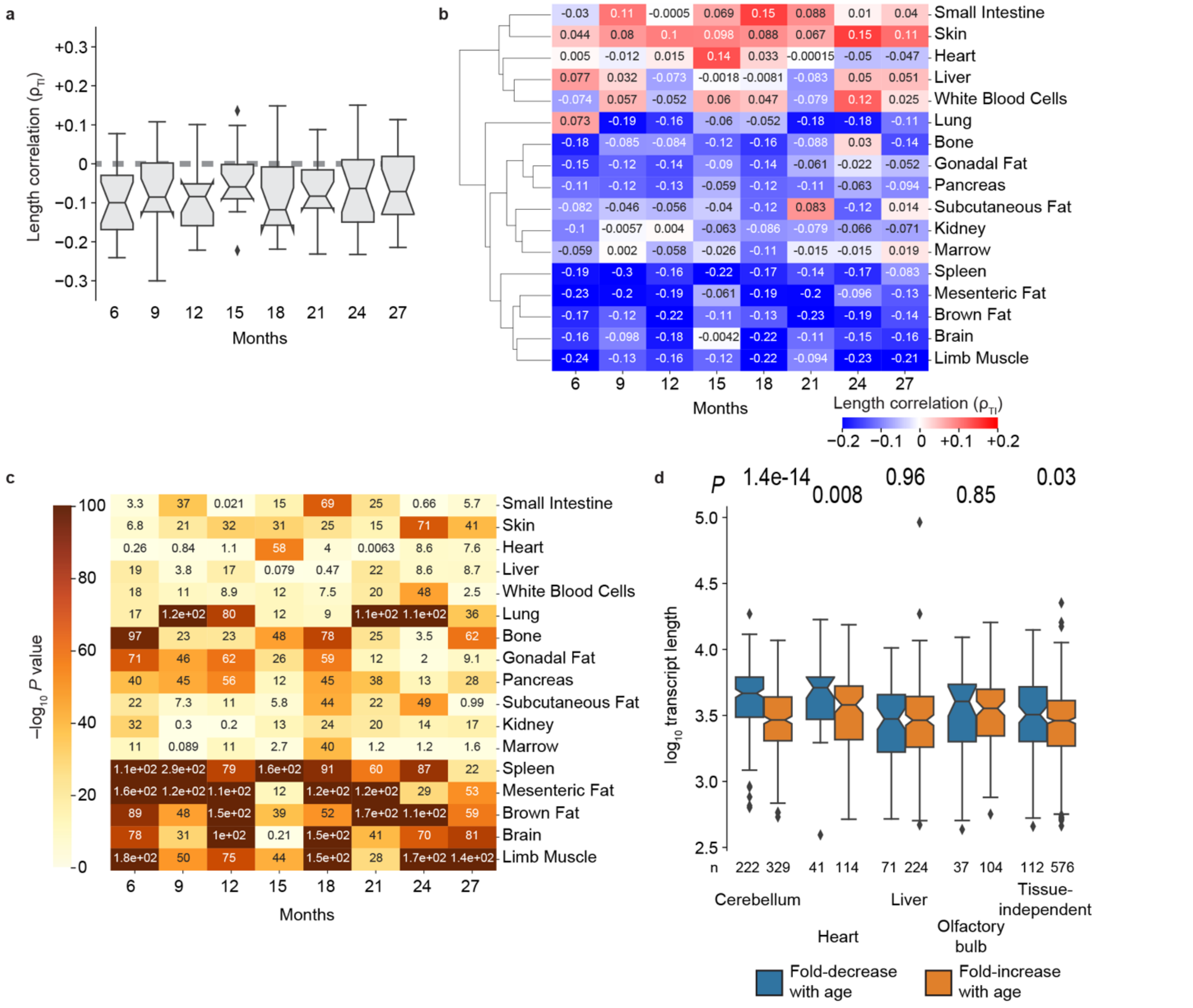

Supplementary Fig. 9. Observation of length-associated transcriptome imbalance in other mouse datasets, related to Fig. 2.

(**a**) Length correlation (ρ_{TI}) relative to 3-month-old mice in Schaum et al. [22] across n=17 tissues per age. (**b**) Length correlation (ρ_{TI}) relative to 3-month-old mice in Schaum et al. [22]. Note how most values indicate negative correlations with length. (**c**) Significance of correlations given in panel **b**. Note how most values indicate P values below 10^{-10}. *P* values were determined using t-distribution for two-sided significance test for Spearman correlation[66] (**d**) Median transcript length for mouse genes reported to be differentially expressed by Benayoun et al., who for 3, 12- and 29-month-old mice solely reported aggregate statistics rather than comparison between individual ages assessed by them[20]. n indicates n=number of genes. *P* values are from two-sided Mann-Whitney *U* test. (**a,d**) In boxplots center is median, notches bootstrapped 95% confidence interval of median, bounds of box 25% and 75% percentiles, whiskers extend height of box 1.5 times, minima and maxima observed minima and maxima.

Supplementary Fig. 10

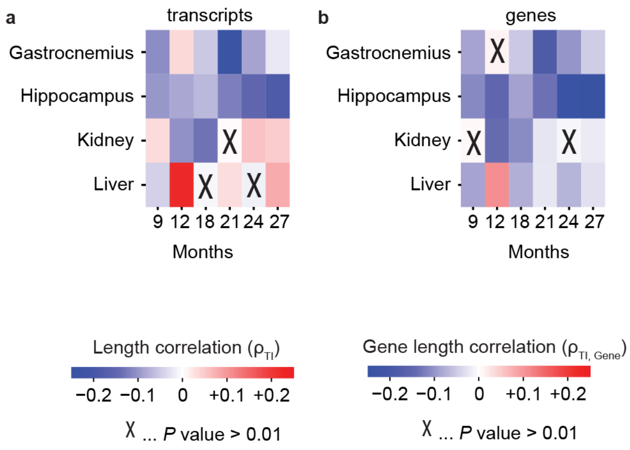

Supplementary Fig. 10. Observation of length-associated transcriptome imbalance in rats, related to Fig. 2.

Spearman correlation between **(a)** transcript and **(b)** gene lengths and relative fold-changes (ρ_{TI}) versus 6-month-old rats on data from Shavlakadze et al. [21]. Note how the majority of values indicate negative correlations with length. (**a**,**b**) *P* values were determined t-distribution based significance of two-sided Spearman correlation[66]

Supplementary Fig. 11

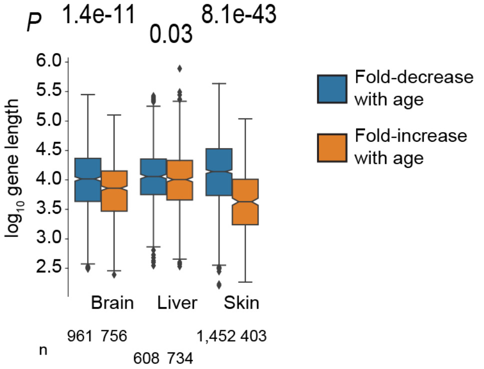

Supplementary Fig. 11. Observation of length-associated transcriptome imbalance in killifish, related to Fig. 2.

Median gene length for killifish genes reported to be differentially expressed between 5- and 39-weeks of age by Reichwald et al. [19]. Gene lengths are as reported by Reichwald et al. [19]. n indicates number of genes. *P* values are from two-sided Mann-Whitney *U* test. In boxplots center is median, notches bootstrapped 95% confidence interval of median, bounds of box 25% and 75% percentiles, whiskers extend height of box 1.5 times, minima and maxima observed minima and maxima.

Supplementary Fig. 12

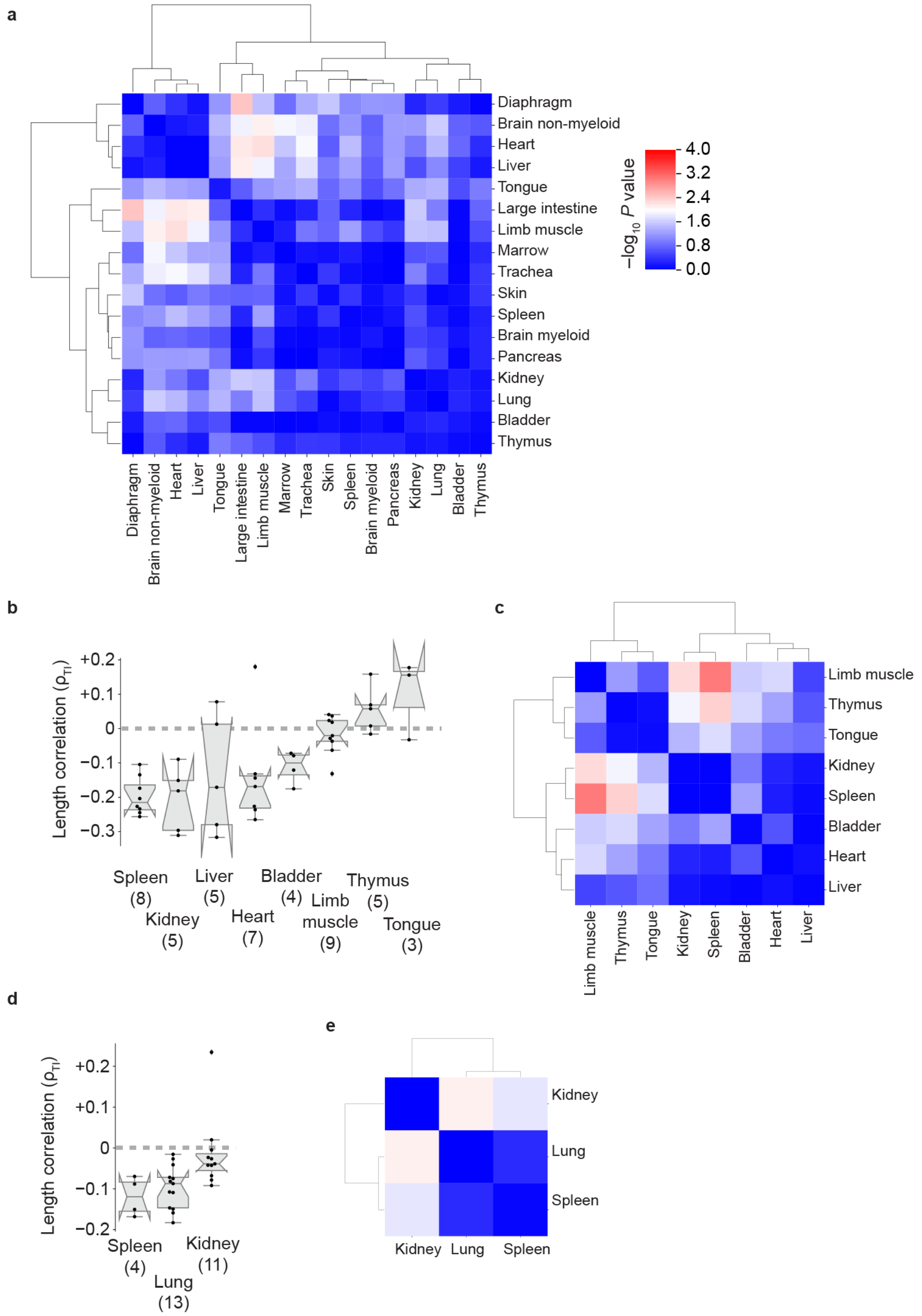

Supplementary Fig. 12. Differences across tissues according to median length correlation of cell types, related to Fig. 2.

(**a**) Clustermap showing *P* values – estimated by two-sided Mann-Whitney *U* test – for cell types of one tissue in Tabula Muris Senis[24] having cell types with a different length correlation (ρ_{TI}) in FACS sorted data. Note how most cell types do not show statistically significant differences (pink color) in their length correlation. (**b**) Boxplot of length correlations (ρ_{TI}) between

transcript length and relative fold-changes for the different cell types reported for different tissues in Tabula Muris Senis' droplet-based approach. (**c**) Clustermap showing *P* values – estimated by two-sided Mann-Whitney *U* test – for cell types of one tissue in Tabula Muris Senis[24] having cell types with a different length correlation (ρ_{TI}) in droplet based data. Note how most cell types do not show statistically significant differences (pink color) in their length correlation. (**d**) Boxplot of calculated Spearman correlation (ρ_{TI}) between transcript length and relative fold-changes for the different cell types reported for different tissues in Kimmel et al. [23]. Number in brackets indicates n=number of different cell types. (**e**) Clustermap showing *P* values – estimated by two-sided Mann-Whitney *U* test – for cell types of one tissue in Kimmel et al. [23] having cell types with a different length correlation (ρ_{TI}). (**b, d**) In boxplots center is median, notches bootstrapped 95% confidence interval of median, bounds of box 25% and 75% percentiles, whiskers extend height of box 1.5 times, minima and maxima observed minima and maxima. Black dots show individual cell types.

Supplementary Fig. 13

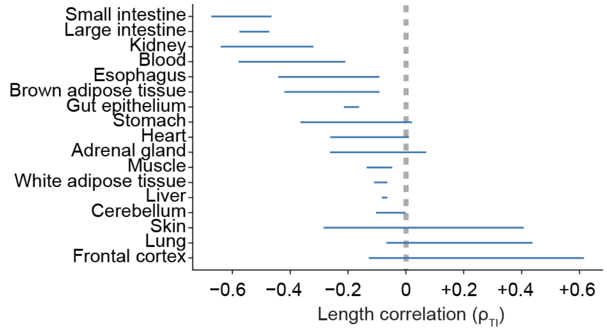

Supplementary Fig. 13. Ranking of tissues according to length correlation of transcriptomic relative fold-change in 24-month-old animals, related to Fig. 2.

Tissues are ranked according to the length correlation (ρ_{TI}) in 24-month-old animals. To convey biological variability, we separately analyze two cohorts of 3 mice that had been sacrificed on different days, and determine the length correlation for each cohort. The horizontal blue lines connect the values obtained for each of the two cohorts for each tissue. Dashed brown line indicates absence of any correlation. Be aware that ranking can slightly differ from ranking using all six mice (Extended Data Fig. 4) due to technical reasons of RNA-seq and differences in the number and identity of detected transcripts. Note that differences between tissues generally are smaller than differences between cohorts.

Supplementary Fig. 14

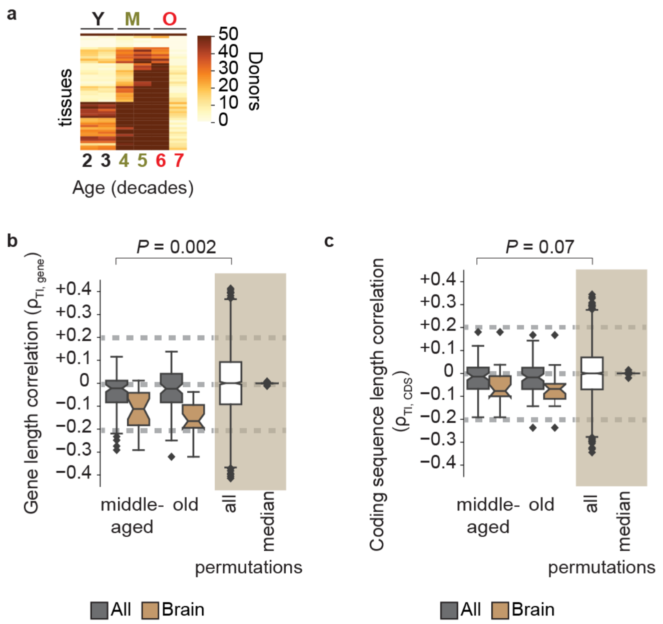

Supplementary Fig. 14. Human tissues show a length-associated transcriptome imbalance during aging, related to Fig. 3.

(**a**) Number of samples archived by the GTEx consortium for individual tissues as function of donor age. “Y” marks young donors aged 20–39 years; “M” marks middle-aged donors aged 40–59 years; “O” marks old donors aged 60–79 years. (**b)** Boxplot representation of the correlation between transcript gene and age-dependent relative fold-changes ($\rho_{TI,\ gene}$) obtained from age comparisons across all 43 tissues of female donors and all 43 tissues of male donors (dark grey) and across 12 brain tissues of female donors and 12 brain tissues of male donors (brown) considered. To provide a baseline for individual sample variability, on the right (shaded in beige), we show the boxplot of values of $\rho_{TI,\ gene}$ obtained from calculating relative fold-changes across all permutations of assigning same age samples to two groups. Under “all,” we show all 2198 permutations obtained from tissues of male and female donors in 20-29- and 30-39-year age brackets. Permutations separated samples into two groups of up 6 randomly chosen from each age groups and tissue and then asked for the length association in the resulting relative fold-changes between those groups. Under “median,” we show the 38 median length-associated changes for each unique tissue with permutations across female or male donors. n= median ρ_{TI} across all combinations of tissues and genders. In boxplots center is median, notches bootstrapped 95% confidence interval of median, bounds of box 25% and 75% percentiles, whiskers extend height of box 1.5 times, minima and maxima observed minima and maxima. *P* values are from two-sided Mann-Whitney U test. (**c**) As panel **b**, but considering the median length of the coding sequences of encoded proteins instead of gene length.

Supplementary Fig. 15

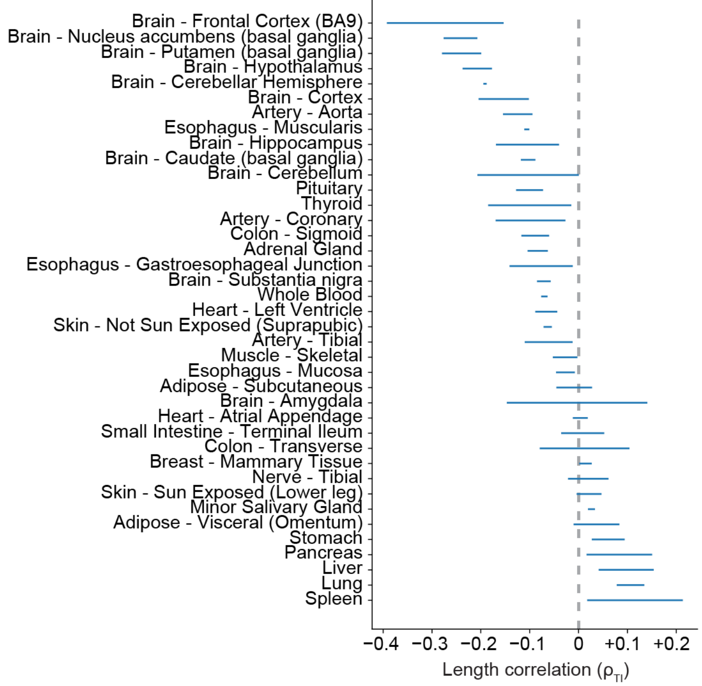

Supplementary Fig. 15. Ranking of human tissues according to strength of length correlation, related to Fig. 3.

Length correlation in individual human tissues for old donors (60-79 years) compared against young donors (20-39 years). Error bars show span between male and female donors. Note that less than 43 tissues are shown as tissues sampled by GTEx differ between male and female donors. Note that differences between tissues often are smaller than differences between male and female donors.

Supplementary Fig. 16

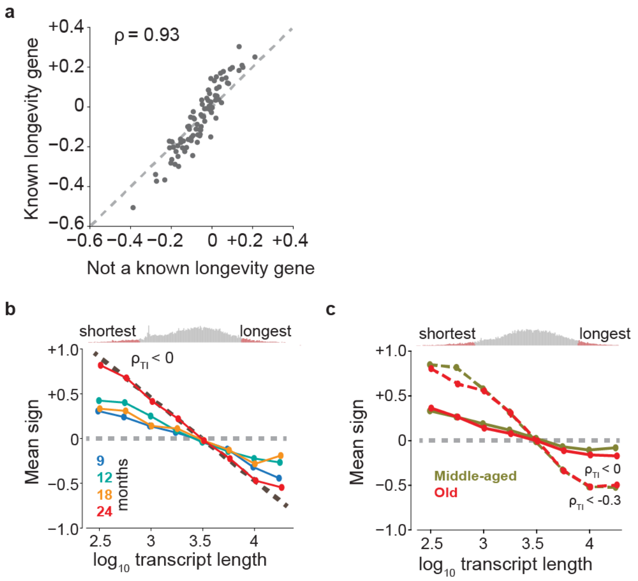

Supplementary Fig. 16. Length-associated transcriptome imbalance of reported longevity-affecting genes, related to Fig. 5.
(a) Scatter plot of correlations between transcript length and age-dependent relative fold-changes in 43 tissues of 60–79-year-old female and male donors (ρ_{TI}) among human genes whose orthologs are known to affect longevity (y-axis) and other human genes (x-axis). Genes that have been reported to affect longevity in a model organism have a similar length-associated transcriptome imbalance within human tissues as genes that have not been reported to affect longevity. (**b**) Average direction of relative fold-change within a tissue for transcripts of a given length for older mice relative to 4-month-old mice. An average of +1 would indicate that all genes with similar transcript lengths show a relative fold-increase, whereas an average of −1 would indicate that all genes with similar transcript lengths show a relative fold-decrease. Colors indicate the ages of the mice compared to 4-month-old mice. Circles show median values of relative fold-change across all samples and error bars represent 95% confidence intervals. Dashed brown line is a guide for the eye that approximates dependence for 24-month-old animals. (**c**) Direction of age-dependent relative fold-change of transcripts, analogous to panel **b**, but for humans. Middle-aged refers to 40–59-year-old donors compared to 20–39-year-old donors, and old refers to 60–79-year-old donors compared to 20-39-year-old donors. The dotted curve highlights samples with strong imbalance ($\rho_{TI} < -0.3$). The shortest and longest genes are the most affected by transcriptome imbalance. In panels **b,c**, plot at the top shows frequency of genes with indicated transcript length. Genes with the 5% shortest and 5% longest median lengths are colored in red.

Supplementary Fig. 17

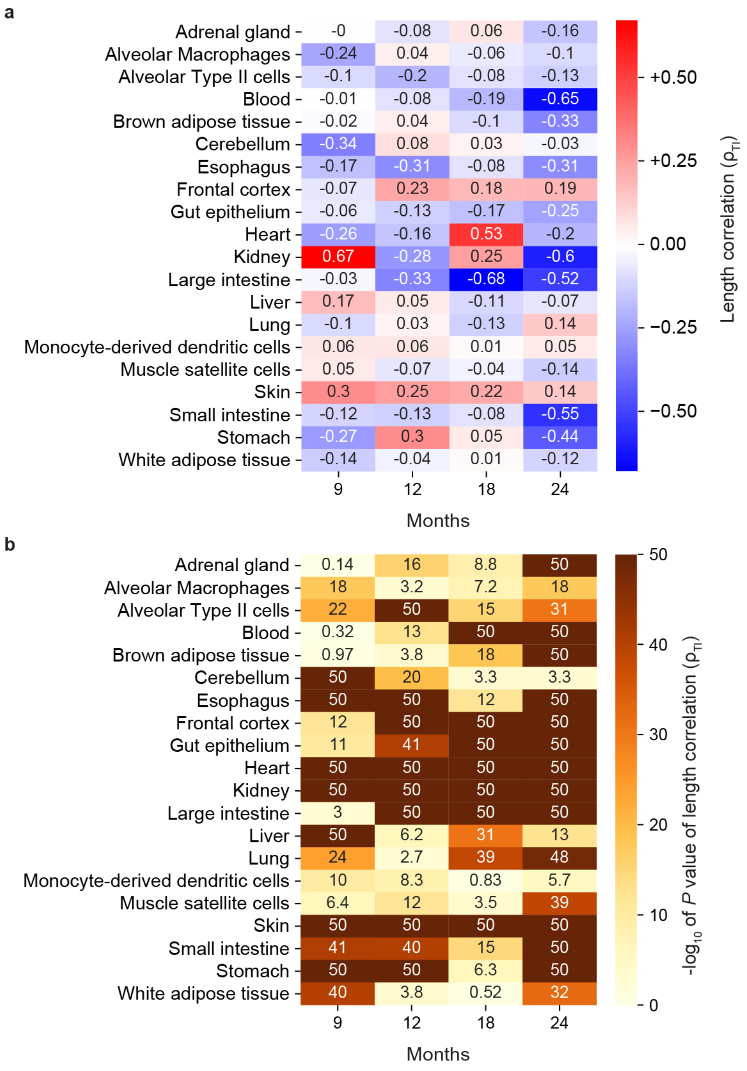

Supplementary Fig. 17. Length-associated transcriptome imbalance for individual tissue-age pairs, related to Fig. 1.

(**a**) Length correlations (ρ_{Tl}) are defined as Spearman correlation between median transcript length and relative fold-changes observed during aging. Note how most length correlations are negative. (**b**) Significance of correlation of panel **a**. Displayed significance values are cropped at 10^{-50}. Note how most P values are lower than 10^{-20}. Significance is determined using t-distribution for two-sided significance test for Spearman correlation[66]. Note that in contrast to the original analysis presented in Extended Data Fig. 4, Alveolar Macrophages, and Alveolar Type 2 cells and Monocyte-derived dendritic cells are included.
