# Supplementary material for: Aging is associated with a systemic length-associated transcriptome imbalance
Source: Nat Aging. 2022 Dec 9;2(12):1191–206. doi: 10.1038/s43587-022-00317-6 (PMC10154227; doi:10.1038/s43587-022-00317-6)
Supplement: Supplementary file 2 — Reporting Summary [file 43587_2022_317_MOESM2_ESM.pdf]

nature portfolio

Corresponding author(s): Thomas Stoeger, Richard Morimoto, Alexander Misharin, Scott Budinger, Luis Amaral

Last updated by author(s): 2022/10/13

Reporting Summary

Nature Portfolio wishes to improve the reproducibility of the work that we publish. This form provides structure for consistency and transparency in reporting. For further information on Nature Portfolio policies, see our Editorial Policies and the Editorial Policy Checklist.

Statistics

For all statistical analyses, confirm that the following items are present in the figure legend, table legend, main text, or Methods section.

n/a	Confirmed	
☐	☒	The exact sample size (*n*) for each experimental group/condition, given as a discrete number and unit of measurement
☐	☒	A statement on whether measurements were taken from distinct samples or whether the same sample was measured repeatedly
☐	☒	The statistical test(s) used AND whether they are one- or two-sided<br>*Only common tests should be described solely by name; describe more complex techniques in the Methods section.*
☐	☒	A description of all covariates tested
☐	☒	A description of any assumptions or corrections, such as tests of normality and adjustment for multiple comparisons
☐	☒	A full description of the statistical parameters including central tendency (e.g. means) or other basic estimates (e.g. regression coefficient) AND variation (e.g. standard deviation) or associated estimates of uncertainty (e.g. confidence intervals)
☒	☐	For null hypothesis testing, the test statistic (e.g. *F*, *t*, *r*) with confidence intervals, effect sizes, degrees of freedom and *P* value noted<br>*Give P values as exact values whenever suitable.*
☒	☐	For Bayesian analysis, information on the choice of priors and Markov chain Monte Carlo settings
☒	☐	For hierarchical and complex designs, identification of the appropriate level for tests and full reporting of outcomes
☐	☒	Estimates of effect sizes (e.g. Cohen's *d*, Pearson's *r*), indicating how they were calculated

Our web collection on statistics for biologists contains articles on many of the points above.

Software and code

Policy information about availability of computer code

Data collection	nextflow 19.10.0; Bcl2fastq 2.17.1.14; Trimmomatic 0.36;Tophat 2.1.0; Tophat 2.1.0; DESeq2 1.20 / 1.22; Singularity 3.2.1-1; STAR 2.6.1d; featureCounts 1.6.4; Samstools 1.6; nf-core 1.4.2; edgeR 3.26.5; Rsubread 1.32.4; nSolver version 4.0.70 , https://github.com/NUPulmonary/stoeger_et_al_2022_transcriptome_imbalance
Data analysis	scipy.stats 1.2.1; Daniel Soper's Free Statistics Calculators 4.0

For manuscripts utilizing custom algorithms or software that are central to the research but not yet described in published literature, software must be made available to editors and reviewers. We strongly encourage code deposition in a community repository (e.g. GitHub). See the Nature Portfolio guidelines for submitting code & software for further information.

Data

Policy information about availability of data

All manuscripts must include a data availability statement. This statement should provide the following information, where applicable:

- Accession codes, unique identifiers, or web links for publicly available datasets
- A description of any restrictions on data availability
- For clinical datasets or third party data, please ensure that the statement adheres to our policy

RNA sequencing data created during this study, and used for Fig. 1 has been deposited under GSE141252. Data underlying other figures has been generated by other research groups and is available from them and/or their respective publications.

Externally generated data can be obtained can be obtained from the following resources listed according to their order of usage:

March 2021

Gallego Romero et al. (https://doi.org/10.1186/1741-7007-12-42 ; Additional file 11), Schaum et al. (https://figshare.com/articles/Differential_Gene_Expression/12227531), Benayoun et al (https://doi.org/10.1101/gr.240093.118 ; Supplementary Table 4), Shavlakadze et al. (https://doi.org/10.1016/j.celrep.2019.08.043; Supplementary Table 1), Reichwald et al. (https://doi.org/10.1016/j.cell.2015.10.071; Supplementary data 4), Kimmel et al. (http://mca.research.calicolabs.com), Tabula Muris Senis (https://figshare.com/articles/Processed_files_to_use_with_scanpy_/8273102 and https://figshare.com/articles/Processed_files_to_use_with_scanpy_/8273102), GTEx (https://gtexportal.org/home/datasets Version 7; dbGaP Accession phs000424.v7); Martinez-Nunez et al. (https://doi.org/10.1093/nar/gkw1109; Supplementary Data file);
Flynn et al. (GSE48043); Mattson et al. (https://doi.org/10.1007/s00251-014-0790-5; Table S2);
Amador-Noguez et al. (https://doi.org/10.1111/j.1474-9728.2004.00125.x; Table S1 for Ames Dwarf mice and Table S2 for Little mice), Ng et al. (https://doi.org/10.1177%2F1559325819876780; Table S8), Murray et al. (https://doi.org/10.1038/s41598-020-64170-6; Supplemental table S1A, S1B); Luizon et al. (https://doi.org/10.1371/journal.pgen.1006449; S1 Table); Hofmann et al. (https://doi.org/10.1016/j.cell.2014.12.016; Supplement 11); Dembic et al. (https://doi.org/10.1016/j.ymgme.2018.10.004; appendix); Selman et al. (https://doi.org/10.1126/science.1177221; Supplemental tables S2A, S2B, S3A, S3B, S3A, S4B);
Hoffman et al. (https://dx.doi.org/10.18632%2Faging.102822; Supplementary table 5);
Jochems et al. (https://doi.org/10.1016/j.celrep.2021.109441; Table S2); Lu et al. (https://doi.org/10.1038/s41586-020-2975-4; Source Data Fig. 4).

Additional data used in this study:

Gene Transcription Regulation Database version 18.06 (http://gtrd.biouml.org:8888/downloads/18.06/); miRDB version 5.0 (http://mirdb.org/download/miRDB_v5.0_prediction_result.txt.gz)

Genes and transcript sequences from Genbank (GRCh38.p10 for human, and GRCm38.p5 for mice) (ftp://ftp.ncbi.nlm.nih.gov/genomes)

GTExPortal version 7 (https://www.gtexportal.org/home/datasets)

Exons from Biomart, using human genome GRCh38.p12 and mouse genome GRCm38.p6 (https://www.ensembl.org/biomart/)

HAGR29,88,89, specifically Longevity Map Build 3 and GenAge Build 19 (https://genomics.senescence.info).

Homologene, version 68 (https://ftp.ncbi.nlm.nih.gov/pub/HomoloGene).

Gene Ontologies using the mapping to NCBI provided by the National Library of Medicine (https://ftp.ncbi.nlm.nih.gov/gene/DATA/gene2go.gz)

Field-specific reporting

Please select the one below that is the best fit for your research. If you are not sure, read the appropriate sections before making your selection.

☒ Life sciences ☐ Behavioural & social sciences ☐ Ecological, evolutionary & environmental sciences

For a reference copy of the document with all sections, see nature.com/documents/nr-reporting-summary-flat.pdf

Life sciences study design

All studies must disclose on these points even when the disclosure is negative.

Sample size

No statistical methods were used to pre-determine sample sizes, but our sample sizes are similar to those reported in previous publications.

The same size of 3 animals per tissue and age was chosen so to allow dissection and preparation of all 17 tissues of interest. We performed the experiment with two different cohorts of mice sacrificed on different days to provide upper bounds for the effect sizes that can be explained by our study (See Supplementary Fig. 3). No sample size calculation was performed. While we can not exclude some false-negative findings, our analysis focused on the identification of phenomena that are detectable within this sample size. We show in Fig. 2 and 3 that the inferred phenomenon also exists in other datasets on aging.

Data exclusions

No data were excluded from the analyses, except for additional control analysis which tested the robustness of the conclusion against different exclusion criteria (Supplementary Fig. 10 c-f).

After completion of the manuscript, we however noted that the original experiment contained muscle tissues for which no sequence data was obtained, and that the original preparation of sequence data included sorted Alveolar Macrophages, and Alveolar Type 2 cells and Monocyte-derived dendritic cells. Preceding the analysis started in this manuscript, sequence data of the latter had not been carried forward toward analysis as quality control metrics appeared different and indicative of lower quality than the other experimental preparations. Retrospectively, analyzing these three cell populations toward a length-associated transcriptome imbalance after the (otherwise) completion of this manuscript, we find – consistent with our comprehensive reanalysis of cell types through public single cell transcriptomic data – a length-associated transcriptome with a relative fold-reduction of long transcripts in Alveolar Macrophages and Alveolar Type 2 cells (Supplementary Fig. 17).

Replication

We considered datasets prepared and/or analyzed by other investigators (Figs. 2, 3 and corresponding supplements). For NanoString analysis we preformed a single experiment. For experiments performed by different laboratories we do not know how often they replicated their analysis. The latter should have little effect on the meta-analysis described by us as we show that within their own analysis a length-association had been present.

Randomization	The different groups correspond to different ages.
Blinding	Investigators were not blinded to group allocation during data collection and outcome assessment and further data analysis. Blinding during data collection was not possible as old mice look different from younger mice; Blinding was not relevant during outcome assessment and further data analysis, as the latter used a machine learning strategy to find the properties informing on age-dependent change.

Reporting for specific materials, systems and methods

We require information from authors about some types of materials, experimental systems and methods used in many studies. Here, indicate whether each material, system or method listed is relevant to your study. If you are not sure if a list item applies to your research, read the appropriate section before selecting a response.

Materials & experimental systems

n/a	Involved in the study
☒	☐ Antibodies
☒	☐ Eukaryotic cell lines
☒	☐ Palaeontology and archaeology
☐	☒ Animals and other organisms
☒	☐ Human research participants
☒	☐ Clinical data
☒	☐ Dual use research of concern

Methods

n/a	Involved in the study
☒	☐ ChIP-seq
☒	☐ Flow cytometry
☒	☐ MRI-based neuroimaging

Animals and other organisms

Policy information about studies involving animals; ARRIVE guidelines recommended for reporting animal research

Laboratory animals	Male C57BL/6J mice were provided by NIA NIH. Six mice were sacrificed at 4, 9, 12, 18, 24 months of age. Animals were house under 14 hour light, 10 hour dark cycle, at 18-23°C with 40-60% humidity.
Wild animals	no wild animals were used in the study
Field-collected samples	no field-collected samples were used in the study
Ethics oversight	The study was approved by Northwestern IACUC, study #IS00014451.

Note that full information on the approval of the study protocol must also be provided in the manuscript.
